# Multiscale Modeling of Germinal Center Recapitulates the Temporal Transition From Memory B Cells to Plasma Cells Differentiation as Regulated by Antigen Affinity-Based Tfh Cell Help

**DOI:** 10.3389/fimmu.2020.620716

**Published:** 2021-02-05

**Authors:** Elena Merino Tejero, Danial Lashgari, Rodrigo García-Valiente, Xuefeng Gao, Fabien Crauste, Philippe A. Robert, Michael Meyer-Hermann, María Rodríguez Martínez, S. Marieke van Ham, Jeroen E. J. Guikema, Huub Hoefsloot, Antoine H. C. van Kampen

**Affiliations:** ^1^ Bioinformatics Laboratory, Epidemiology and Data Science, Amsterdam Public Health Research Institute, Amsterdam Institute for Infection and Immunity, Amsterdam, Netherlands; ^2^ Department of Hematology and Oncology, International Cancer Center, Shenzhen University General Hospital, Shenzhen University Health Science Center, Shenzhen, China; ^3^ Université de Paris, CNRS, MAP5 UMR 8145, Paris, France; ^4^ Department for Systems Immunology and Braunschweig Integrated Centre of Systems Biology, Helmholtz Centre for Infection Research, Braunschweig, Germany; ^5^ Institute for Biochemistry, Biotechnology and Bioinformatics, Technische Universität Braunschweig, Braunschweig, Germany; ^6^ IBM Research Europe, Zürich, Switzerland; ^7^ Department of Immunopathology, Sanquin Research and Landsteiner Laboratory, Amsterdam UMC, University of Amsterdam, Amsterdam, Netherlands; ^8^ Swammerdam Institute for Life Sciences, University of Amsterdam, Amsterdam, Netherlands; ^9^ Department of Pathology, Lymphoma and Myeloma Center Amsterdam (LYMMCARE), Amsterdam University Medical Centers, Amsterdam, Netherlands; ^10^ Biosystems Data Analysis, Swammerdam Institute for Life Sciences, University of Amsterdam, Amsterdam, Netherlands

**Keywords:** multiscale model, plasma cell differentiation, T follicular helper cell, CD40 signaling, germinal center

## Abstract

Germinal centers play a key role in the adaptive immune system since they are able to produce memory B cells and plasma cells that produce high affinity antibodies for an effective immune protection. The mechanisms underlying cell-fate decisions are not well understood but asymmetric division of antigen, B-cell receptor affinity, interactions between B-cells and T follicular helper cells (triggering CD40 signaling), and regulatory interactions of transcription factors have all been proposed to play a role. In addition, a temporal switch from memory B-cell to plasma cell differentiation during the germinal center reaction has been shown. To investigate if antigen affinity-based Tfh cell help recapitulates the temporal switch we implemented a multiscale model that integrates cellular interactions with a core gene regulatory network comprising BCL6, IRF4, and BLIMP1. Using this model we show that affinity-based CD40 signaling in combination with asymmetric division of B-cells result in switch from memory B-cell to plasma cell generation during the course of the germinal center reaction. We also show that cell fate division is unlikely to be (solely) based on asymmetric division of Ag but that BLIMP1 is a more important factor. Altogether, our model enables to test the influence of molecular modulations of the CD40 signaling pathway on the production of germinal center output cells.

## Introduction

Germinal centers (GCs) are anatomical structures located inside B-cell follicles within secondary lymphoid organs that play an important role in the adaptive immune system (1, 2). Through subsequent rounds of cell proliferation, somatic hypermutation (SHM) and positive selection the B-cell receptor (BcR) is optimized for antigen (Ag) binding in a process called affinity maturation. This eventually results in the development of memory B-cells (MBCs) and plasma cells (PCs) that produce high affinity antibodies (Abs), which provide an effective immune protection. GCs comprise two functional zones. In the dark zone (DZ) centroblasts (CBs) rapidly proliferate and accumulate SHMs in the genes that encode their BcR. The light zone (LZ) is mainly characterized by the presence of centrocytes (CCs), follicular dendritic cells (FDCs) that present Ag in the form of immune complexes to GC B cells (3), and T follicular helper (Tfh) cells. CCs capture and internalize Ag through their BcR in an affinity-dependent manner triggering survival signals. Subsequently, the Ag is processed by the CCs resulting in class II MHC-peptide complexes (pMHCII) presented to the Tfh cells. Hence, B cells compete in an affinity-dependent way for interaction with Tfh cells, facilitating CD40 and cytokine signaling to become positively selected. Positively selected CCs may return to the CB state and recycle to the DZ to undergo further rounds of proliferation and SHM. Alternatively, positively selected CCs may differentiate to MBCs or PCs (4–8). Recently, it was also shown that GC B-cell migration influences PC development (9) The cellular and molecular mechanisms that regulate PC and MBC differentiation remain largely unknown, while such knowledge would crucially advance our understanding of GC-associated diseases such as B-cell lymphomas and autoimmune disorders. In this research we present a multiscale computational model (MSM) integrating molecular and cellular mechanisms to investigate PC differentiation.

*In vivo* studies in which Tfh-dependent positive selection of CCs was triggered in a BcR-independent fashion using the DEC205 surface lectin indicated that the interaction of CCs with Tfh cells critically determines positive selection and subsequent generation of PCs (10, 11) Other studies suggested that BcR signaling, but not Tfh interaction, initiates PC differentiation (12–15). The role of BcR signaling in PC differentiation is supported by the observation that long-lived PCs in bone marrow produce high-affinity Abs that contain many SHMs (13, 16–18). Smith and co-workers showed that the extend of affinity maturation of MBCs and PCs differs for NP hapten-specific B-cell responses typically resulting in high-affinity PCs and low-affinity MBCs (18). Other work suggested a temporal switch during the GC reaction resulting in the production of MBCs primarily during the early GC phase while long-lived bone-marrow (BM) PCs are generated at later stages (19). In support, Shinnakasu and co-workers showed that lower affinity cells at earlier stages of the GC reaction are favored to enter the MBC compartment and also suggested that high-affinity GC B cells are preferentially selected to enter the cell-cycle and undergo PC differentiation (20).

Previously, an agent-based model (ABM) was developed that assumes that all CCs positively selected by Tfh cells subsequently recycle to the DZ for further proliferation, mutation and differentiation (2). Experimental evidence for this model was in part provided by demonstrating PC precursors in the DZ (13, 21, 22). This computational model was dubbed LEDA (LEave the GC through the DArk zone) and distributes the captured Ag asymmetrically during cell division to the daughter cells. The Ag-retaining cells differentiate into PCs and leave the GC (2). Other models were investigated in this paper, such as LEDAX, in which the decision about differentiation is already taken during the interaction with Tfh cells in the LZ irrespective of asymmetric division. A probabilistic decision is made after symmetric division in the DZ to decide if the B-cell differentiates to an output cell or heads for another round of selection. Nevertheless, we wanted to test the effect of asymmetric division on PC differentiation and, therefore, we used the LEDA model as a starting point. However, direct experimental evidence that asymmetric division determines cell fate is lacking.

A large body of research focuses on the molecular mechanisms underlying PC and MBC differentiation including epigenetics (23–25), the role of various transcription factors (TFs), and gene regulatory networks (GRN) [e.g., (26–29)]. Our MSM is built on a core GRN comprising three TFs (BCL6, IRF4, and BLIMP1) that are directly involved in PC differentiation. The TF B lymphocyte induced maturation protein 1 (BLIMP1) is essential for PC differentiation and regulates a large number of target genes required for the function of these cells (30). For example, BLIMP1 represses class II transactivator (CIITA) and activation-induced cytidine deaminase (AID), thereby inhibiting Ag presentation and GC associated AID-dependent Ig gene diversifications, respectively (30, 31). BLIMP1, however, may not initiate PC differentiation which has been suggested to start by down-regulation of the Paired Box 5 (PAX5) and B-cell lymphoma 6 (BCL6) proteins, which supports the theory that BcR signaling, resulting from BcR – Ag interaction, initiates this process (13). BcR signaling results in the repression of BCL6 (32), which is an important factor for BcR diversification and sustained cell proliferation. However, other studies have shown that Interferon regulatory factor 4 (IRF4) initiates plasmablast (PB) differentiation by inducing expression of BLIMP1 (33, 34). This does not exclude the possibility that BcR signals are involved in increasing IRF4 levels. CD40 signaling, resulting from CC – Tfh interaction, upregulates IRF4, which subsequently activates BLIMP1 and leads to PC differentiation. In PCs, IRF4 can also bind to its own promoter to create a positive feedback mechanism that maintains high IRF4 expression and, consequently, BLIMP1 expression (35). BLIMP1 is generally considered to repress gene expression but it may also induce gene expression of IRF4 and other genes (30). BCL6 is highly expressed in GC B cells and inhibits both the expression of BLIMP1 and IRF4. BCL6 binds to its own promoter to inhibit its own transcription thereby resulting in an autoregulatory negative feedback loop (36). In turn, BLIMP1 and IRF4 repress BCL6, which is down-regulated in PC differentiation.

It is challenging to integrate the cellular and molecular mechanisms involved in PC differentiation since details about the effect of cellular interactions through signaling on the underlying molecular networks are not known in full detail. Conversely, the effect of GRN states on cell behavior or phenotype also remains to be elucidated in more detail. Moreover, these mechanisms operate at different time scales. One way of proceeding is to model (affinity dependent) signals resulting from cellular interactions that affect the underlying GRN, which in turn determines cell fate. We present a MSM integrating molecular and cellular mechanisms to investigate PC differentiation. In particular, we integrate two pre-existing and published computational models: the cell-based LEDA model (2) and a differential equation-based GRN including BCL6, IRF4, and BLIMP1 (37). This GRN model considers BcR and CD40 signals delivered to the B cells but it assumes that only the CD40 signal initiates and progresses differentiation. Other (cytokine-driven) signals during B/T-cell interactions are neglected in our model. Our MSM integrates and investigates asymmetric division and (affinity-based) CD40 signaling in PC differentiation.

Using this model we show that affinity-based CD40 signaling in combination with asymmetric division result in MBC and PC generation in accordance with the temporal switch. In contrast, a constant strong CD40 signal does only result in PCs, while a constant weak signal results in MBC output throughout the GC reaction. We also conclude that cell fate division is unlikely to be (solely) based on asymmetric division of Ag since this does not result in the differentiation of all high-level BLIMP1 B-cells. Vice versa, PCs differentiated on the basis of high BLIMP1 levels are a mixture of cells with and without internalized Ag indicating that not only Ag retaining cells engage in differentiation. We propose experiments to validate our computational findings. Altogether, our model enables to test the influence of molecular modulations of CD40 signaling pathway onto the production of MBC and PCs.

## Methods

### Computational Model at the Cellular Level

**Figure 1:**
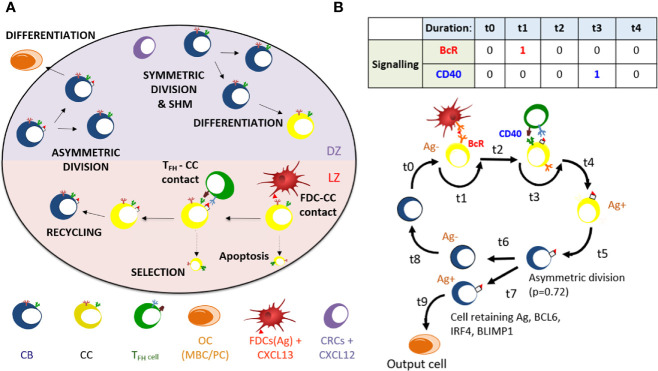
**(A)** Overview of cellular processes in the ABM. In an established GC a dark zone (DZ) and a light zone (LZ) are distinguished. CBs and CCs prefer to move in the direction of the CXCL12 and CXCL13 chemokines produced by the CRCs and FDCs respectively. Tfh cells prefer to move towards the LZ. FDCs carry Ag that can be captured by CCs. CCs may be positively selected through interaction with Tfh cells after which they can recycle to the dark. In the DZ the CB will (a)symmetrically divide. After cell division, an output cell is produced, or the cell differentiates to a CC. Cells die through apoptosis if they do not interact with the FDC and Tfh cells. **(B)** Schematic overview of the BcR and CD40 signaling events during the GC reaction. Durations *t* indicate non-fixed time intervals (cell states). At the end of each interval the concentrations of BCL6, IRF4, and BLIMP1 are updated using the differential equations. A CB (Ag−; blue cell) differentiates to a CC (Ag−; yellow cell) within a time duration *t0*. The CC interacts with the FDC for a time duration *t1* during which BcR signaling occurs. Subsequently, CD40 signaling is active for duration *t3* during B-cell – Tfh interaction. Successful interaction will result in an Ag+ cell. Asymmetric division occurs with a probability p=0.72. Differentiation of CB to a CC always initializes the CC to Ag−.

The MSM that we developed is an extension of the pre-existing “hyphasma” model, which is a detailed ABM of the GC that simulates the behavior of individual GC cells and their interactions (**Figure 1A**) (2, 38). Under the so-called LEDA hypothesis it assumes that output cells exit the GC from the DZ after asymmetric division. Here, we summarize the relevant aspects of this model. The model simulates a GC reaction for 21 days (504 h) at a time resolution of 0.002 h (7.2 s). Parameters for the ABM in our simulations are provided as **Supplementary Files** (parameters 1–5.txt). The GC is represented as a three-dimensional sphere of grid points with N=64 grid points in each direction (lattice constant = 5 µm). This grid hosts agents that represent CCs, CBs, Tfh cells, and FDCs. In addition, pre-calculated gradients of CXCL12 and CXCL13 chemokines are imposed on this grid. Originally, the ABM was initiated with a fixed number of three founder B cells (2). However, in our simulations we assumed a continuous influx of, on average, 2 cells per hour in the first 96 h resulting in approximately 100 founder cells accounting for the observation that early GCs are highly polyclonal (39, 40). The behavior in terms of division, differentiation, interaction, and cell death between these cells is defined by a set of rules. CBs, CCs and Tfh cells move according to persistent random-walk based on chemokine gradients, while FDCs have a fixed position on the grid. The affinity of the BcR is defined as the Manhattan distance (L1 norm) between the BcR and the Ag within a four dimensional “shape space” (41, 42). This distance represents the minimum number of SHMs required for the BcR to acquire maximum affinity for the Ag. Hence, the BcR sequence is not explicitly encoded but rather the shape space position of a B-cell determines its BcR affinity. SHM moves the BcR one step in the shape space thereby increasing or decreasing the distance to the Ag, which is converted to an affinity value between 0 and 1 using a Gaussian weight function. The discrete nature of the shape space translates to 25 discrete affinity values (**Supplementary Figures 1** and **2**).

In the model, B cells (CBs) proliferate in the DZ while accumulating BcR mutations, and migrate as CCs to the LZ where they can interact with FDCs to capture Ag with a rate depending on the BcR affinity. This provides survival signals to the CCs and rescues them from apoptosis. Higher affinity CCs will capture more Ag and, subsequently, will outcompete lower affinity CCs for Tfh interaction to become positively selected. If the Tfh interacts with many B cells at a time it will signal to the one with highest amount of internalized Ag. The positively selected CCs are recycled into CBs and migrate to the DZ for further rounds of division and SHM. The number of divisions of recycled CBs depends on the amount of captured Ag during the selection process. During cell division the Ag is asymmetrically distributed in 72% of the cell divisions (43). Daughter cells that receive the captured Ag differentiate to output cells after one or more divisions and exit the GC. Daughter cells that did not receive Ag cycle back to the LZ as CCs. Daughter cells of CBs that divide symmetrically receive half of the Ag and both become CCs.

### Computational Model at the Molecular Level

**Figure 2:**
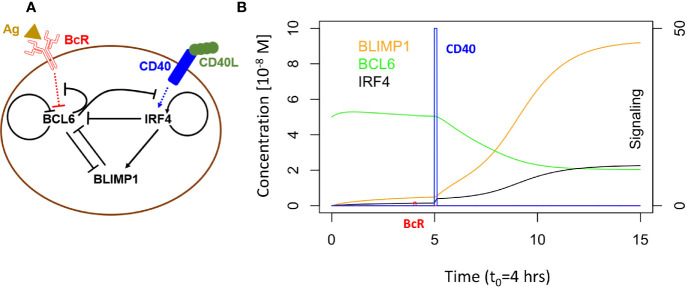
**(A)** B-cell with GRN and signaling events. Arrows indicate activation. Bar-headed lines denote inhibition. BCL6 is inhibited upon binding of the Ag to the BcR. IRF4 is activated upon binding of CD40L to CD40 during Tfh – B-cell interaction. **(B)** GRN temporal dynamics upon binding of Ag and CD40L. Each time unit represents 4 h. The protein levels of BCL6 (blue), IRF4 (black), and BLIMP1 (orange) have units of 10^−8^ M and are shown over an interval of 60 h. The BcR signal (red) and CD40 signal (green) are present for a short duration (*t1* and *t3* in **Figure 1B**). BcR signaling results in a slight temporary change in TF concentrations. In contrast, CD40 signaling results in a switch of all TF levels going from a B-cell to a PC (BLIMP1+) phenotype (in approximately 40 h in this example). CD40 signal intensity in the multiscale model varies between 0 and 50. BcR signal is fixed to 1.

Martinez and co-workers developed a computational model representing a core GRN involved in PC differentiation (**Figure 2A**) (37). This model includes three TFs, i.e., BCL6, BLIMP1 and IRF4 that are modeled by ordinary differential equations (ODEs). The level of these genes is controlled by the BcR and CD40 signals (**Supplementary Information**, Equations 1 to 5; **Supplementary Table 1** lists the parameter values for the model). This GRN represents a bistable system with one state (BCL6 high, BLIMP1/IRF4 low) denoting the CBs/CCs and a second state (BLIMP1/IRF4 high, BCL6 low) representing PCs (**Figure 2B**).

GC B cells integrate upstream signals from BcR and CD40 signaling pathways. When a BcR signal is induced through binding with the Ag, then BCL6 is degraded. However, its level is rapidly restored to the initial steady state (BCL6 high) when the signal is removed (unbinding of Ag). The CD40 signal induced during interaction with a Tfh cell increases transcription of IRF4 which in turn increases the level of BLIMP1. This results in the PC phenotype (BLIMP1+) This state is irreversible due to the positive autoregulatory feedback of IRF4 and the cooperative binding of the TFs to the DNA.

### Multiscale Model

**Figure 3:**
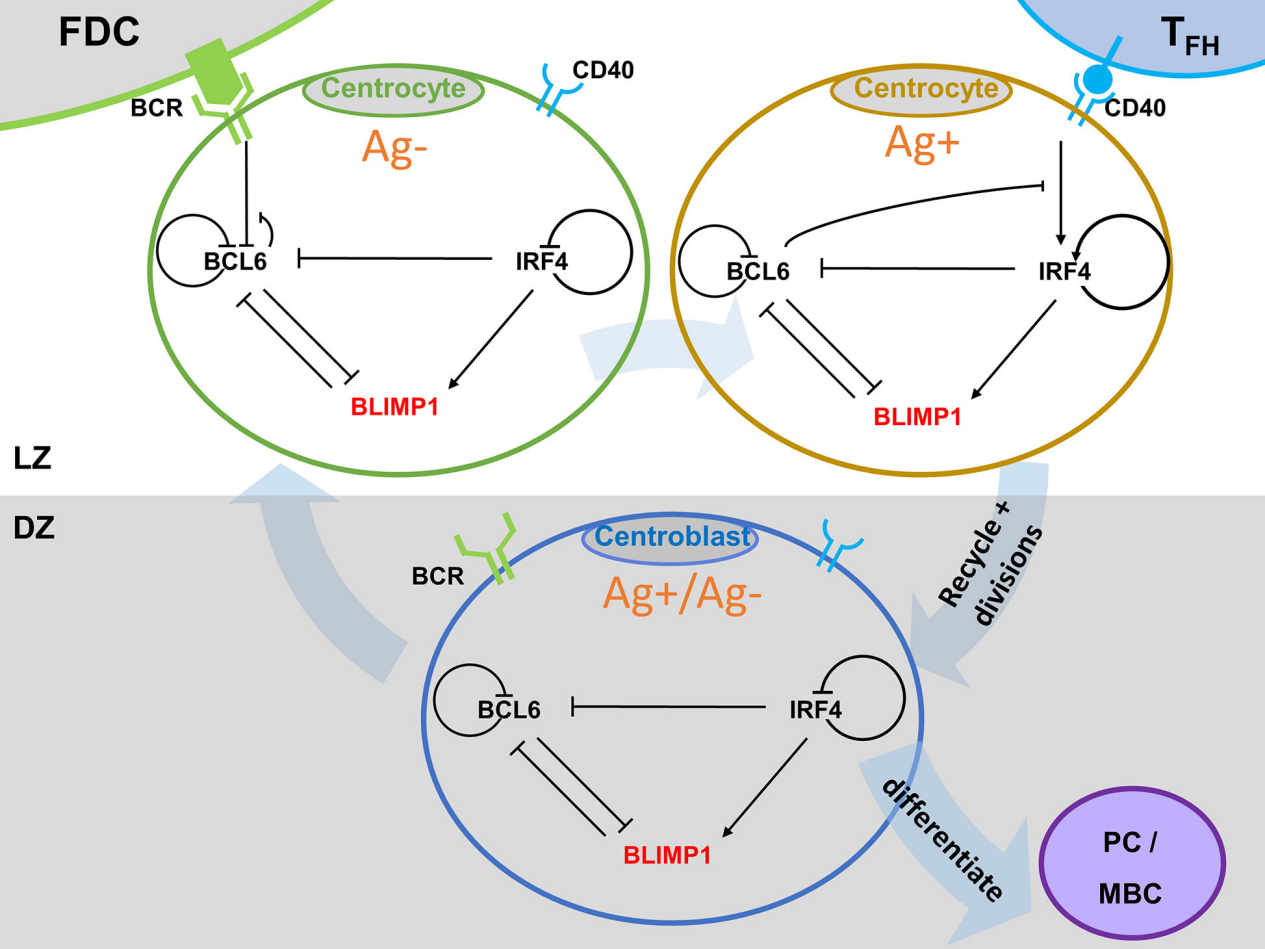
Multiscale model for PC differentiation. The cellular model (ABM) and molecular GRN (ODE) models are integrated by embedding the GRN in each B-cell and output cell of the ABM. Signals through the BcR (FDC interaction) and CD40 (Tfh interaction) change the state (TF concentrations) of the GRN which is updated at every time step of the ABM. A positively selected CC becomes Ag+ by definition. In scenario 1 simulations an Ag+ cell differentiates to an output cell after asymmetric cell division. In scenario 2, sufficient CD40 signaling may increase BLIMP1 levels to obtain a PC phenotype (BLIMP1+). For precise cell type definitions see **Table 2** and **Supplementary Figures 6–8, 20**.

To enable the investigation of cellular and molecular mechanisms involved in PC differentiation we integrated the ABM and the GRN through the embedding of the GRN (set of ODEs) in each individual B-cell and output cell of the ABM (**Figure 3**). This was achieved by adding additional properties (ODEs (initial) TF levels, and BcR/CD40 signal) to each agent (cell) of the ABM.

Founder cells and daughter cells resulting cell division are initialized with the same initial concentrations for BCL6, IRF4, and BLIMP1. The cell-based ABM and the GRN model operate at different time scales, e.g., weeks and hours respectively. Consequently, the relatively fast changes in the GRN affect the longer term outcome on the cell level. This is accomplished by updating the TF concentrations at every time step (7.2 s) of the ABM while taking into account transcription and degradation rates, and using the levels of the TFs of the previous time point as initial values for the ODEs. If a CC binds to the FDC (**Figure 1B**) it receives a constant BcR signal (bcr0 = 1 in the ODE) for the duration *t1* of binding. If the CC binds to a Tfh cell it will receive a CD40 signal (see below) for the duration *t3* of binding. BcR and CD40 signals never occur simultaneously because binding to the Tfh cell only occurs after detaching from the FDC.

It has been shown that TFs may distribute unequally in daughter CBs after division (44). Consequently, it has been hypothesized that asymmetric division may affect cell fate. Therefore, the MSM allows for asymmetric division of both Ag and the TFs with a probability of 0.72 (2, 43). Following asymmetric division, the TF concentrations become zero in one daughter cell while the other daughter cell assumes the concentration from the parent cell. In a symmetric division the TF and Ag concentrations in both daughter cells are set to half the concentrations of the parent cell.

### Tfh Facilitated CD40 Signaling

The MSM considers a constant or an affinity-based CD40 signal by defining cd40 (see **Supplementary Information Equations 1–5; Supplementary Table 1**). The magnitude of the constant signal was set to 50 to ensure that after Tfh contact the BLIMP1 level of the B-cell sufficiently increases to eventually differentiate to a PC while also maintaining typical GC dynamics (e.g., CB and CC cell count profiles). For the affinity-based signal we assume that higher affinity B cells capture more Ag and present more pMHCII to Tfh cells resulting in an increased Tfh – B-cell interaction and, therefore, an increased CD40 signaling. The affinity-based CD40 signal was defined by setting cd40=affinity*50. Since affinity assumes values between 0 and 1, the CD40 signal has a strength between 0 and 50. This ensures that at maximum affinity the B-cell will always differentiate into a PC while at lower affinities MBCs will be produced (see below). Note that in simulations 3 and 4 (see below) higher values of the CD40 signal results in PC differentiation even after symmetric division (which reduces the BLIMP1 level by 50%) because the BLIMP1 level will rapidly return to its high-level equilibrium value due to the positive autoregulatory feedback of IRF4 that also remains at a relatively high level (**Supplementary Figures 3–5**).

### Simulations

**Table 1** shows the five simulations that were performed. The parameters for each simulation are provided as **Supplementary Files**. Scenario 1 simulations 1 and 2 represent a model in which asymmetric division of Ag determines cell fate. The Ag-retaining daughter cell (Ag+) differentiates to an output cell. In these simulations we tracked the CD40 signaling and the levels of the TFs but the GRN does not affect the fate of the B-cell and, therefore, does not affect the outcome of the simulation. However, after completion of scenario 1 simulations we inspect the BLIMP1 level of the output cells to define a PC and MBC subset (see cell definitions below and **Supplementary Figure 20**). Scenario 2 simulations 3, 4, and 5 represent the model in which we use the BLIMP1 level to decide on cell fate. In these simulations cells with a high BLIMP1 concentration will differentiate to PCs regardless of the Ag state (Ag+ or Ag−) of the cell. For both cell-fate decision rules we compare results obtained with a constant and affinity-based CD40 signal. In simulation 5 we use a constant CD40 signaling with cd40 = 10. All simulations are terminated after 21 days. In the result section we present the outcome of these 5 individual simulations. However, we also repeated simulation 3 and 4 30 times with different random seeds, which shows that the amount of variability observed in the temporal dynamics (**Supplementary Figures 16–19**) is limited. Also the resulting variability in the reported percentages is very low (standard error <0.01, most standard deviations <1%; **Supplementary Tables 3** and **4**). Scenario 1 simulations were not repeated but a similar amount of variability is expected.

**Table 1:** Definition of simulations.

		CD40 signal	
		Constant	Affinity-based
Decision rule for differentiation	**Ag inheritance** (scenario 1)	Simulation 1 (CD40 = 50)	Simulation 2
	**BLIMP1 level** (scenario 2)	Simulation 3 (CD40 = 50)Simulation 5 (CD40 = 10)	Simulation 4

### Definition of (output) Cells

**Table 2** shows the definition of cell types in scenario 1 (Ag+ decision rule) and scenario 2 (BLIMP1+ decision rule) simulations. **Supplementary Figures 6 to 8** and **20** provide further explanation. In scenario 2, we do not explicitly discriminate between MBCs and PCs but define “output” cells solely on the basis of its Ag status, i.e., the daughter cells that retains the Ag after asymmetric division (Ag+ cell) differentiates to an output cell (2). In a post-simulation step we use the BLIMP1 level to classify the output cells to PCs (Ag+ and BLIMP1+; ≥ 8 × 10^−8^ M) and MBCs (Ag+ and BLIMP1−; <8 × 10^−8^ M). We have defined MBCs in this way because a BLIMP1− cell does not represent a PC while in this model an Ag+ cell was defined as an output cell. Although this is not an ideal MBC definition it correctly recapitulates the MBC dynamics as observed in Weisel and co-workers (19). In the MSM a recycled CB is, by definition, Ag+ and goes through one or multiple rounds of divisions prior to differentiation to an output cell. Consequently, Ag+ cells represent a mixture of recycled CBs, dividing cells, and output cells. In scenario 1, dividing Ag+ cells that have the potential to become a PC (i.e., Ag+/BLIMP1+) are annoted as PBs to allow a further discrimination between cell states in the model. In scenario 1, Ag− output cells are non-existent by definition and, hence, all Ag− cells are CBs or CCs. In scenario 2, cells may become a PC if they are BLIMP1+ irrespective of its Ag status and, consequently, PCs may either be Ag+ or Ag−. BLIMP1+ cells that are not (yet) output cells are annotated as PB (Ag+ or Ag−). In scenario 2, Ag+/BLIMP1− output cells are considered to be MBCs.

**Table 2:** Definition of cell types based on Ag status and BLIMP1.

		Scenario 1		Scenario 2	
		BLIMP1+	BLIMP1−	BLIMP1+	BLIMP1−
Output cell	**Ag+**	PC	MBC	PC	MBC
Not output cell	**Ag+**	PB	CB	PB	CB
Output cell	**Ag**−	NA	NA	PC	NA
Not output cell	**Ag**−	CB/CC	CB/CC	PB	CB/CC

NA, not applicable.

### Software

The MSM was implemented in C++ and simulations were done on a MacOS Mojave 10.14.5 operation system. Run times of a single simulation take approximately 8 h on a single core of an Intel Core i7 processor. Model repetitions were carried out on the Dutch national e-infrastructure with the support of SURF Cooperative (www.surfsara.nl). Output files of the simulation were analyzed in R (Core Team, 2019) version 3.5.3 using various libraries: forcats (0.5.0), purr (0.3.4), tidyr (1.0.3), tibble (3.0.1), ggplot(2_3.3.0), tidyverse (1.3.0), viridis (0.5.1), viridisLite (0.3.0), ggnewscale (0.4.1), readr (1.3.1), dplyr (0.8.5). The MSM is available from GitHub (https://github.com/EDS-Bioinformatics-Laboratory/MSM_PCdifferentiation).

## Results

### Ag Inheritance-Based GC Output with Constant and Strong CD40 Signal Exclusively Produces PCs (Scenario 1)

**Figure 4:**
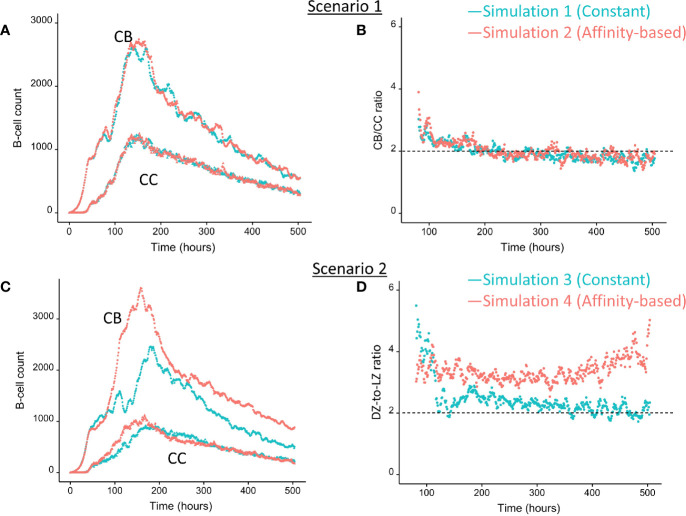
Overall GC dynamics with constant CD40 signal (CD40 = 50; blue) and affinity-based CD40 signal (red). **(A)** Scenario 1. Number of CBs (top curves) and CCs (bottom curves). **(B)** Scenario 1. DZ-to-LZ ratio calculated from CB and CC counts. **(C)** Scenario 2. Number of CBs (top curves) and CCs (bottom curves). **(D)** Scenario 2. DZ-to-LZ ratio calculated from CB and CC counts.

**Figure 5:**
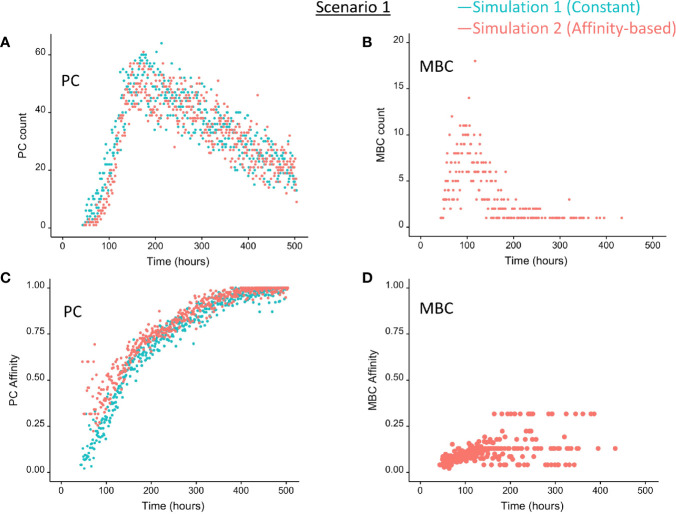
Output cells for scenario 1 simulations with a constant CD40 signal (CD40 = 50; blue) or affinity-based CD40 signal (red). **(A)** Number of PCs, **(B)** number of MBCs, **(C)** PC affinity, and **(D)** MBC affinity during GC reaction. Post-simulation inspection of BLIMP1 levels of the output cells (Ag+) allows to discriminate between PCs (Ag+/BLIMP1+) and MBCs (Ag+/BLIMP1−). No MBCs are produced with a constant CD40 signal.

We wondered how the levels of BLIMP1 compared to internalized-Ag status (Ag+ or Ag−) in GC B-cell population when CD40 signal was constant and strong. This served as a reference for scenario 2 simulations (**Table 1**). The scenario 1 model is based on the hypothesis that Ag-retaining (Ag+) cells differentiate to a mixture of PC and MBC output cells. This theory in which asymmetric division drives PC differentiation resulting in PCs from the earliest stages of the GC reaction seems incompatible with the experimentally observed temporal switch (19). **Figure 4A** shows the overall dynamics of simulation 1. The CB and CC counts show a typical GC response with the total cell count approximating about 3800 cells at 142 h (6 days). **Figure 4B** shows the DZ-to-LZ ratio, which fluctuates around 2 in agreement with *in vivo* experiments (11). **Figure 5A** shows the number of PCs during the GC reaction, which by definition emerge from the very initial stages of the GC reaction. **Figure 5C** shows that the affinity of these PCs increases during the course of the GC reaction.

**Figure 6:**
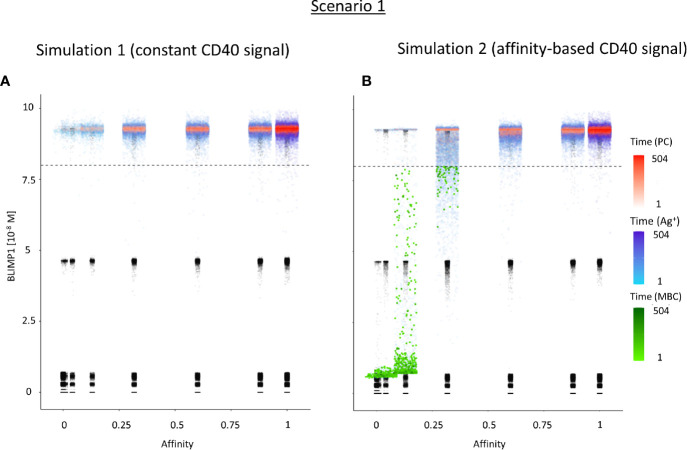
Distribution of PCs, MBCs, and Ag+ (but not PC/MBC) cells with respect to their affinity, BLIMP1 level, and time of generation for scenario 1 simulations. **(A)** Simulation 1 with a constant CD40 signal (CD40 = 50). All output cells are PCs (no MBCs are generated). **(B)** Simulation 2 with an affinity-based CD40 signal. Output cells are mainly MBCs during the early GC reaction for B cells with affinity levels between 0 and 0.25. As the GC reaction progresses and B-cell affinity increases, BLIMP1 levels consequently rise above the threshold leading to a switch in the GC output towards mainly PCs for affinity levels between 0.25 and 1. Each dot represents a cell. Black dots represent cells other than PCs, MBCs, Ag+. Color gradient represents time from 1 to 504 h. Affinity assumes discrete values. Dotted line represents the BLIMP1 threshold (8 × 10^−8^M) for PC differentiation. Intermediate BLIMP1 levels arise mainly due to symmetric division of cells with high BLIMP1 level. Since values are recorded only in case of an event (e.g., cell division, Tfh interaction, differentiation) and because of steep curve of the BLIMP1 profile after Tfh-cell contact, BLIMP1 levels seem restricted to particular values but are not. Low and high BLIMP1 levels represent steady-states. Jittering of affinity values has been applied to prevent too many overlapping data points but causes some overlap of the lower affinity classes.

**Table 3** shows the percentages of (output) cells at day 21 of the simulation. The full tables and cell counts are listed in the **Supplementary File Counts_and_Percentages.xlsx**. Inspection of the BLIMP1 level of the output cells facilitates post-simulation differentiation between PCs (Ag+/BLIMP1+) and MBCs (Ag+/BLIMP1−). During the GC reaction, about 5% (15,136 cells) of all CCs (290,291) differentiate to a PC (Ag+/BLIMP1+) while no MBCs (Ag+/BLIMP1−) are generated because the constant but strong CD40 signaling enforces high BLIMP1 levels for Ag+ cells. A fraction of PB (Ag+/BLIMP1+) cells do not develop into output cells due to symmetric cell division, which generates two Ag− daughter cells (**Supplementary Figure 7**). The subset of Ag− cells (CBs and CCs), which are not output cells nor PBs comprise a mixture of BLIMP1+ and BLIMP1− representing 12 and 62% of all cells respectively. Consequently, an additional maximum of 12% (36,124 cells) could potentially have developed into a PC if BLIMP1 level was considered as a criterion for differentiation. **Figure 6A** shows the distribution of PCs (Ag+/BLIMP1+), PBs (Ag+), and CCs/CBs (Ag−/BLIMP1−, Ag−/BLIMP1+). No MBCs are produced. CCs and CBs are distributed over all affinity classes and have BLIMP1 levels below the threshold (<8 × 10^−8^ M) that defines PCs. PCs (high BLIMP1 level) emerge from the early stages but their number and affinity increases with time. Finally, the figure shows an increasing number of Ag+ cells that increase in affinity over time but do are not output cells. About 79% of the subset of Ag+ cells did not develop into output cells despite their high BLIMP1 levels. In addition, about 17% of the Ag− cells are BLIMP1+.

**Table 3:** Percentages of cell types at day 21.

	Scenario 1		Scenario 2		
	Constant CD40 = 50	Affinity-based	Constant CD40 = 50	Affinity-based	Constant CD40 = 10
PC	5	5	14	13	3
PB	20	19	28	26	18
MBC	0	0.3	0	0.3	2
CB/CC	75	76	58	61	77
	100.0	100.0	100.0	100.0	100.0

In summary, the scenario 1 model with constant CD40 signaling simulation produces PCs of low to high affinities but no MBCs due to the strong CD40 signal. Approximately 75% of the PCs are generated after the peak response of the output cells (**Figure 5A**; **Supplementary Figure 9**) and are of relatively high affinity due to ongoing affinity maturation (**Figure 5C**). A large fraction of the Ag+ cells are BLIMP1+ while most Ag− cells are BLIMP1−. Considering BLIMP1 levels of the Ag− cells, a larger number of PCs should potentially have been generated.

### Ag Inheritance-Based GC Output With a Strong Affinity-Based CD40 Signal Enables the Production of Both PCs and MBCs (Scenario 1)

Since no MBCs *(*Ag+/BLIMP1−) were generated in simulation 1, we wanted to investigate the effect on output cell subsets (post-simulation) when applying an affinity-based CD40 signal to control the levels of BLIMP1. In this simulation (simulation 2), the generation of output cells is still fully determined by Ag inheritance after asymmetric division and, consequently, CD40 signaling nor BLIMP1 level affects the cell fate or GC reaction. Consequently, the overall dynamics of this simulation is approximately the same as for the first simulation (**Figure 4**). Difference in overall dynamics result from stochasticity in the model. **Figures 5A, C** show the number of PCs and corresponding affinity during the GC reaction. **Figures 5B, D** show the number of MBCs (Ag+/BLIMP1−) and affinity respectively. In contrast to simulation 1, low affinity MBCs are generated during the earlier phase of the GC response and generation of PCs seems slightly delayed although stochasticity in the model prevents a firm conclusion. The number of PCs at the end of the GC reaction is similar to simulation 1 (5% of all cells corresponding to 14,303 cells; **Table 3**). In addition, 833 (0.3%) MBCs were generated. The number of PBs, CCs, and CBs is similar to simulation 1. Also in this simulation an additional 35,159 Ag− cells (12% of all cells) could potentially have developed into a PC if the BLIMP1 level was used as a decision rule for PC differentiation during the simulation. **Figure 6B** shows that MBCs are of low affinity, have BLIMP1 levels below the PC threshold (<8 × 10^−8^ M) and are generated during the early phase of the GC response. Increased affinity abolished MBCs as a result of increasing BLIMP1 level resulting in a transition to PCs with BLIMP1 levels above the threshold. We also observe that at affinity=0.25 a larger number of Ag+ cells with intermediate BLIMP1 levels occur, which is a consequence of affinity-based signaling in which cells that have weaker CD40 signaling more slowly increase their BLIMP1 levels. About 75% of the subset of Ag+ cells did not develop into output cells despite high BLIMP1 levels. In addition, about 16% of the Ag− cells are BLIMP1+.

In summary, affinity-based CD40 signaling simulation produces a mixture of early lower affinity MBCs followed by later higher affinity PCs. Approximately 76% of the PCs are generated after the peak response of the output cells (**Figure 5A**) while 85% of the MBCs are produced prior to the peak response (**Figure 5B**; **Supplementary Figure 11**). This temporal shift is in agreement with recent findings (19). Overall, we see that a large fraction of Ag+ non-output cells are BLIMP1+ and, therefore, a larger number of PCs should potentially have been generated.

### BLIMP1 and Ag-Defined Fate Decisions Do Not Lead to MBC Generation Under Strong Constant CD40 Signal (Scenario 2)

**Figure 7:**
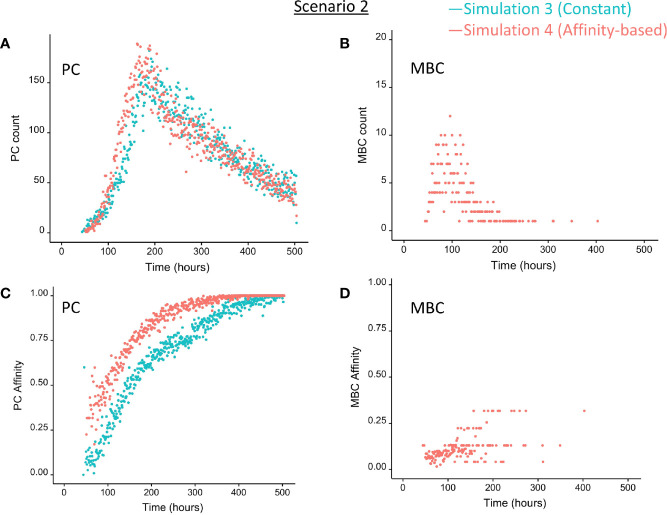
Output cells for scenario 2 simulations with a constant CD40 signal (CD40 = 50; blue) or affinity-based CD40 signal (red). **(A)** Number of PCs (Ag+BLIMP1+, Ag−BLIMP1+), **(B)** number of MBCs (Ag+BLIMP1−), **(C)** PC affinity, and **(D)** MBC affinity during GC reaction. No MBCs are produced with a constant CD40 signal.

**Figure 8:**
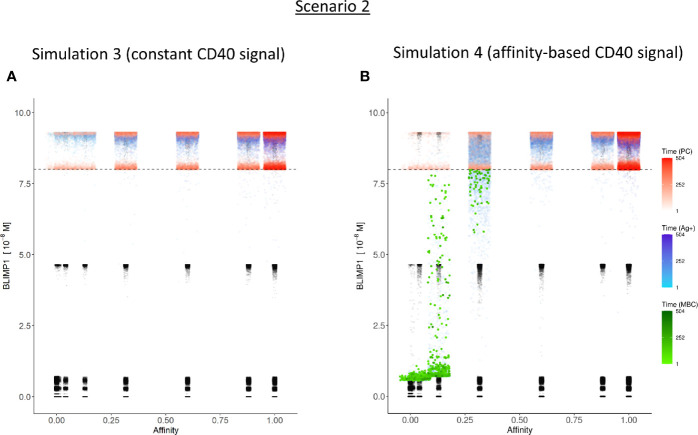
Distribution of PCs, MBCs, and Ag+ (but not PC/MBC) cells with respect to their affinity, BLIMP1 level, and time of generation for scenario 2 simulations. **(A)** Simulation 1 with a Constant CD40 signal (CD40 = 50). All output cells are PCs (no MBCs are generated). **(B)** Simulation 2 with an affinity-based CD40 signaling. Output cells are mainly MBCs during the early GC reaction, which then switches to PC production. For a further description see **Figure 6**. In **(A)** and **(B)** PCs (red) are generated accross all affinity levels.

We then wondered whether we could determine cell fate based on the coupling of BLIMP1 level and Ag status under a strong constant CD40 signal. In this simulation (simulation 3), the generation of PCs is fully based on BLIMP1 levels and does not take Ag status into account, i.e., subsequent to a series of cell divisions the CBs with high BLIMP1 levels (≥8 M) differentiate to PCs (Ag+BLIMP1+ or Ag−BLIMP1+). In addition, Ag-retaining cells with low BLIMP1 levels (<8M) differentiate to MBCs (Ag+BLIMP1−). **Figure 4C** shows the overall GC dynamics, which is similar to scenario 1 simulations but the DZ-to-LZ ratio slightly increased (**Figure 4D**). The effect of stochasticity on the overall GC dynamics and the DZ-to-LZ ratio is limited as shown from repeated simulations in **Supplementary Figures 16** and **17**. **Figures 7A, C** show the number of PCs and corresponding affinity. No MBCs are produced in this simulation due to strong CD40 signaling that enforces high BLIMP1 levels and, consequently, only PCs are generated. This was not surprising considering simulation 1. However, the number of PCs at the end of the GC reaction is about a factor 3 larger compared to simulation 1 (14% of all cells corresponding to 38,684 cells; **Table 3**). The number of PBs is slightly larger compared to the simulation 1 while the number of CBs and CCs are slightly reduced. Approximately 33% of all BLIMP1+ cells (115,310) differentiate to PCs and about two-third of these cells are Ag−. The distribution of PCs, and Ag+ cells (**Figure 8A**) is similar compared to simulation 1 (**Figure 6A**) but PCs now assume BLIMP1 levels ranging from 8 to about 9 while in simulation 1 all Ag+ output cells assumed the highest possible BLIMP1 level (i.e., ~9). The bimodal distribution is observed since some CBs will differentiate immediately when the BLIMP1 level passes the threshold while other cells may engage in one or more cell divisions giving BLIMP1 additional time to reach its maximum value.

In summary, the MSM allows to couple the decision for differentiation based on both BLIMP1 level and Ag status. With a constant strong CD40 signaling the scenario 2 simulation produces only PCs of low to high affinities but no MBCs. Substantially more PCs are generated in comparison to simulation 1 and 72% of these PCs are generated after the peak response of the output cells (**Figure 7A**; **Supplementary Figure 10**), which are of relatively high affinity (**Figure 7C**). The slight increase in DZ-to-LZ ratio implies that the transzone migration rates in scenario 2 are no longer in full agreement with the patterns observed in (11).

### BLIMP1− and Ag-Defined Fate Decisions Under a Strong Affinity-Based CD40 Signal Produce MBCs and Show a Temporal Switch (Scenario 2)

Considering no MBCs were generated under strong constant CD40 signal we wondered whether the decision for differentiation based on BLIMP1 level and Ag status under an affinity-based CD40 signal produces both PCs and MBCs. The overall GC dynamics of this simulation (simulation 4) is shown in **Figure 4C** which are clearly different from simulation 3 in which a constant CD40 signal was used. The number of CCs is similar, but the number of CBs largely increased resulting in an increased DZ-to-LZ ratio to approximately 3 to 4 (**Figure 4D**). The effect of stochasticity in the model on GC dynamics and DZ-to-LZ ratio is shown in **Supplementary Figures 18 and 19** for 30 repetitions. **Figure 7A** shows that the number of PCs in simulation 3 (38,684 cells) and simulation 4 (35,670) is similar but, overall, the PCs have a higher affinity (**Figure 7C**). Affinity-based signaling results in the generation of MBCs of low affinity mostly during the early phase of the GC response (**Figures 7B, C**). The number of PCs at the end of the GC reaction is approximately a factor 2.5 larger compared to simulation 2 that also involved affinity-based signaling (13% of all cells; **Table 3**). The percentage of MBCs (0.3%; 781 cells) is comparable to simulation 2. This corresponds to 0.5% of all BLIMP1− cells. Similar to simulation 3, approximately 33% of all BLIMP1+ cells (107,943) differentiate to a PC and about two-third of these cells are Ag−. The distribution of PCs, MBCs, and Ag+ cells is shown in **Figure 8B**.

In summary, in scenario 2, the affinity-based CD40 signaling simulation produces PCs and a small fraction of MBCs. However, substantially more PCs are generated in comparison to scenario 1. 75% of these PCs are generated after the peak response of the output cells while 89% of the MBCs are produced prior to this peak and are of lower affinity. (**Figure 7**; **Supplementary Figure 12**). Although we now observed a temporal shift there is a significant increase in the DZ-to-LZ ratio indicating transzone migration rates that are not in agreement with (11). We also observed that a substantial fraction of the PCs are Ag− indicating that the decision for PC differentiation should not (fully) be based on Ag status.

### BLIMP- and Ag-Defined Fate Decisions Under Weak Constant CD40 Signal Produce MBCs But Fail to Show a Temporal Switch (Scenario 2)

**Figure 9:**
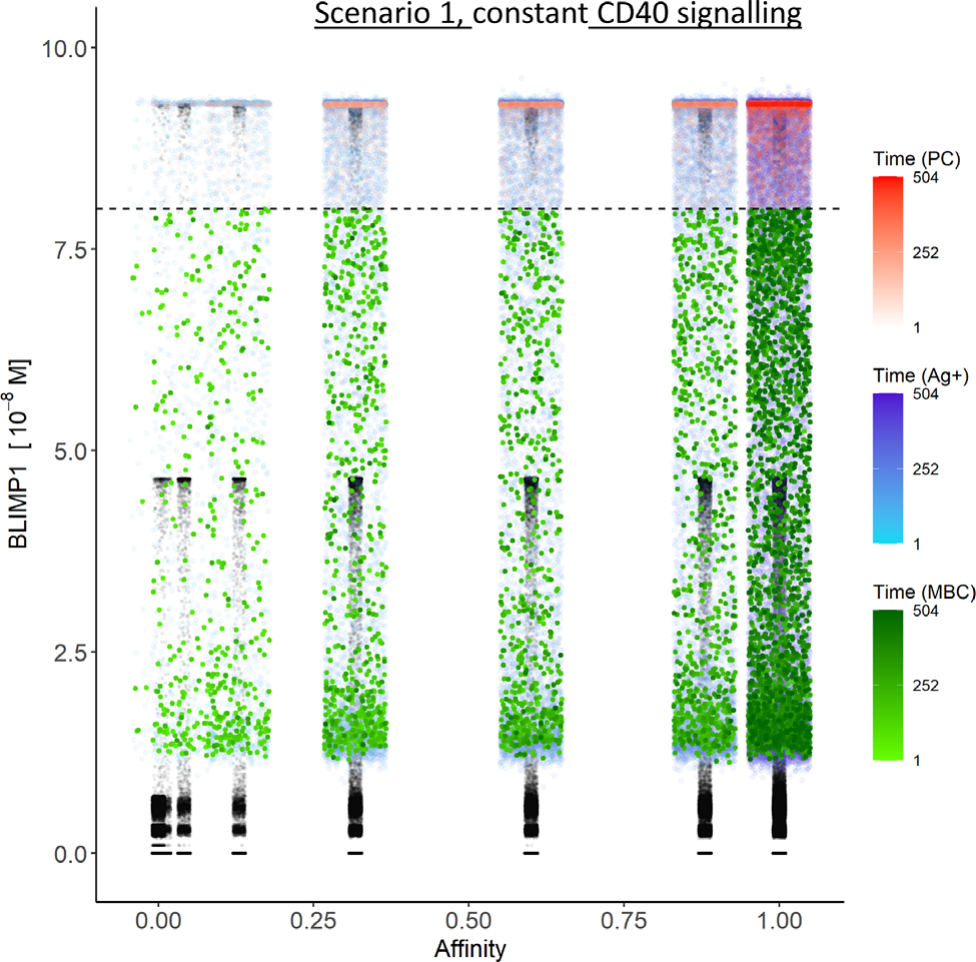
Distribution of PCs, MBCs, and Ag+ (but not PC/MBC) cells with respect to their affinity, BLIMP1 level, and time of generation for scenario 2 simulations. Constant CD40 signaling (CD40 = 10). For a further description see **Figure 6**.

**Figure 10:**
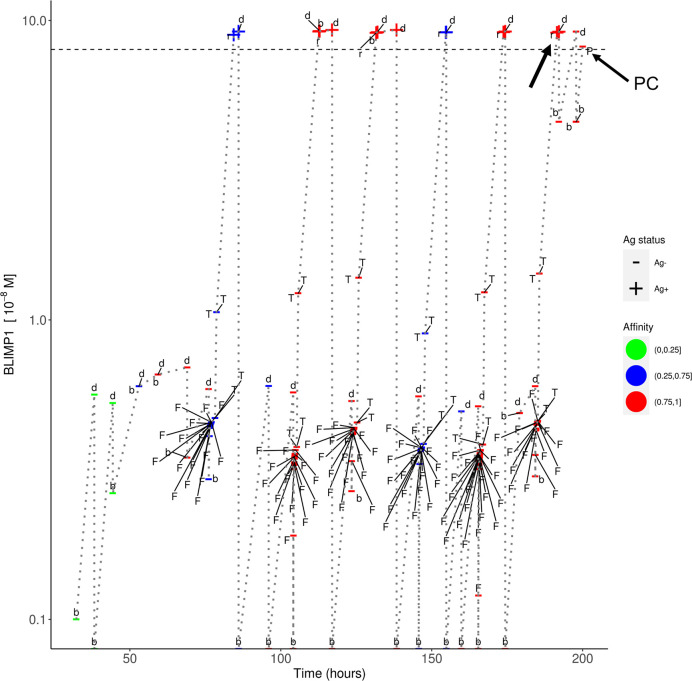
Temporal dynamics of B-cell lineage for scenario 2, affinity-based CD40 signaling (simulation 4). The dotted lines traces the lineage of a single founder B_cell entering at the initial phase of the GC reaction up to a PC differentiation event at about 200 h. At each event (d, division; b, born, F, FDC contact; T, Tfh interaction; r, recycle to DZ; P, PC differentiation) the BLIMP1 level, Ag status (Ag+/Ag−), and affinity (low, medium high) is shown. The horizontal dotted line represents the BLIMP1 threshold for PC differentiation. CBs go through one or more cell divisions (d, b) before differentiating to CCs to have interaction with the FDC and Tfh cells. The affinity of the B-cells in this lineage shows an overall increase although SHM may also decrease affinity (red to blue color). Ag− cells are created from asymmetric division. BLIMP1 level varies in time as a result of transcriptional activity and (a)symmetric division. After interaction with a Tfh cell, the BLIMP1 level increases due to CD40 signaling. Asymmetric division may leave the BLIMP1 level unchanged or reduce it to 0. Symmetric division reduces the concentration with 50%. In this particular lineage we observe that a Ag+BLIMP1+ cell (indicated by the arrow) asymmetrically divides resulting in a Ag−BLIMP1− cell, which subsequently increases its BLIMP1 level again in subsequent divisions, and final differentiates to a PC (Ag−BLIMP1+).

In simulation 3, we used a strong and constant CD40 signal (cd40 = 50) that prevented the generation of MBCs because Tfh cell help will always sufficiently increase the BLIMP1 level to exclusively result in PC differentiation. In contrast in simulation 4 we allowed the CD40 signal to vary with affinity resulting in a temporal switch from MBCs to PCs. Since an constant high-level is not realistic (i.e., no MBCs are produced) we questioned if we could generate both MBCs and PCs by using a constant but lower CD40 signal (cd40 = 10; simulation 5). In this simulation the overall GC dynamics is similar to the other simulations (**Supplementary Figure 14A**). The DZ-to-LZ ratio fluctuates around a value of 2 (**Supplementary Figure 14B**). The total number of cells during the course of the GC reaction is comparable to the other simulations. Compared with simulation 3, a constant and weak CD40 signaling indeed results in the generation of MBCs and even increased five-fold (2%; 5,048 cells) at the expense of a lower number of PCs (3%; 10,204 cells; **Supplementary file Counts_and_Percentages.xlsx**). However, since the CD40 signal strength does not change over time this simulation does not result in a temporal switch but a steady but low production of MBCs throughout the GC reaction (**Supplementary Figure 13**). We also observe that only Ag+BLIMP1+ and no Ag−BLIMP1+ PCs are generated reflecting that cells that divided symmetrically result in daughter cells with BLIMP1 and IRF4 concentrations that are never high enough to return to the BLIMP1+ state. **Figure 9** shows the distribution of the PCs, MBCs and Ag+ cells. About 73% of the PCs are produced after the peak of the output cell production and also the majority of the MBCs (74%) are produced after the peak (**Supplementary Figure 15**). In **Figure 10**, we show an example of the temporal dynamics of B-cell lineage during the GC reaction starting with a founder cell that eventually results in PC differentiation. It shows how BLIMP1 level, Ag status, and affinity evolve as a result of the synergistic interaction between the molecular and cellular level at different events in the MSM.

In summary, a constant and weak CD40 signaling strength is able to produce MBCs throughout the GC reaction at the expense of PCs and, consequently, no temporal switch is observed.

## Discussion

We presented a multiscale computational model integrating cellular and molecular mechanisms, operating at different time scales, to investigate output cell differentiation based on Ag status and/or BLIMP1 level. In this paper we compared these mechanisms for cell-fate determination under various instances of CD40 signaling.

An important insight from our model (simulations 2 and 4) is the observation that regulation of the BLIMP1 level through affinity-dependent but not constant CD40 signaling, results in the occurrence of a temporal transition from MBC to PC output during the GC reaction (19, 45). In addition, simulation 2 showed that the LEDA theory (i.e., a mechanism in which the decision for output cell differentiation is solely based on asymmetric division but not on BLIMP1 level) does not exclude a temporal transition. However, scenario 1 simulations produce BLIMP1+ cells of which approximately 33% are Ag− showing that a decision for differentiation solely based on Ag status is not adequate since this will exclude a large number of BLIMP1+ cells from PC differentiation. Inspection of the PCs (BLIMP1+ cells) of the scenario 2 simulations shows that these are a mixture of Ag+ (~11%) and Ag− (~22%) cells. This also argues against asymmetric inheritance as sole mechanism for PC differentiation. It is known that high affinity GC B cells present more pMHCII molecules to Tfh cells resulting in increased expression of CD40L and hence stronger CD40 signaling which determines cell phenotype (21, 46–49) and results in faster and more cell divisions in the DZ (17, 21, 50, 51).

The lack of experimental data to support our findings is clearly a weakness of our work and complementary experiments are required to validate the results from our simulations. In particular, we propose experiments to generate data about the (1) average number of PCs and MBCs that leave a single GC during its life time; (2) extend and and/or role of (a)symmetric division of Ag and TFs in relation to cell fate; (3) quantitative relationship between BcR affinity, CD40 signaling strength and BLIMP1 level.

One other apparent weakness of the MSM concerns the definition of MBCs as Ag+BLIMP1− cells. Although mechanisms of MBC differentiation are even less understood than for PC differentiation, we needed a route to generate both MBCs and PCs to make the model more realistic. Noticeably, lack of MBCs would have had a (small) effect on the overall GC dynamics. In favor of our approach is the observation that MBCs have indeed low BLIMP1 levels (8) and the observation of a temporal switch with low affinity MBCs and higher affinity PCs. The current definition, however, implies that Ag status (Ag+) is one of the determinants in MBC differentiation and that also MBCs leave the GC through the DZ. However, there is no experimental evidence to support this assumption at this stage. The generation of MBCs could be improved by modeling of the BTB domain and CNC homolog 2 [BACH2; (20, 52)] and the contribution of the CD40 pathway to MBC differentiation. Inclusion of the BACH2 in the GRN is, however, not sufficient as was recently shown in another model (53). One way forward is to model different cell fate (apoptosis, MBC/PC differentiation, and DZ recycling) for different levels of Tfh cell help, and to include MYC, FOXO1, IL-4, and IL-21 (Laidlaw and Cyster (54) Nat Rev Immunol; Luo et al. (55) Immunity]. However, the work of Krautler and co-workers seems to support the conclusion that Tfh-cell acts *via* signals other than CD40. Moreover, the stochastic selection of low-affinity B cells has been proposed as yet another mechanism to produced MBCs (18, 45, 56) or PCs (56–59).

One assumption in the MSM concerns the asymmetric division of TFs. It has been shown that BCL6 and IRF4 may distribute unequally in daughter CBs after division (44, 60), and it has been hypothesized that this may affect cell fate. To the best of our knowledge, neither symmetric nor asymmetric distribution of BLIMP1 during division has been reported.

Results from our simulations show that approximately 15,000 – 35,000 PCs and 800 MBCs are produced in a single GC reaction corresponding to about 5 – 14% and 0.3% respectively of all GC cells. Although data is available regarding numbers of PCs and MBCs generated spleen and bone marrow during an immune response [e.g., Sugimoto-Ishige et al. (61), Kishi et al. (62) J. Imm., 185, 211, Weisel et al. (19)], these numbers always represent percentages of observed PCs/MBCs from total number of splenic or bone marrow cells, which are impossible to translate to number of output cells from a single GC and, therefore, not directly comparable with our results.

In a recent study, it was shown that both BcR signaling and help from Tfh cells are required for positive selection of CCs, as signaling pathways that emanate from the BcR and CD40 ligation are rewired in GC B cells. In contrast to naïve B cells, GC B cells require both signals to induce the c-Myc transcription factor, which is a critical mediator of GC B-cell survival, cell-cycle reentry, and a marker of positive selection (55). These results indicate that CCs compete for Tfh-cell help in a BcR affinity-dependent fashion. It also has been proposed that c-Myc^+^Bcl6^lo^IRF4^+^ cells are most likely PC precursors while Myc^+^Bcl6^hi^IRF4^−^ will recycle to the DZ (7). However, cells with low BCL6 and higher IRF4 or BLIMP1 expression have also been found in the DZ, which supports the recycling model our MSM (13, 21). In support for our model, it has been shown that DZ B cells displayed a more prominent PC gene signature than LZ B cells (12, 13). Similarly, high-affinity LZ B cells showed a strong PC signature including a high expression of IRF4 in high-affinity CCs. Their experiments indicated that PC differentiation is initiated by signals delivered to high-affinity B cells in the LZ with subsequent transition to a late PC phenotype occurring after migration to the DZ.

These and other, sometimes contradicting studies, on MBC and PC differentiation clearly show the need for additional research to unravel mechanisms underlying cell-fate decisions in the GC. Further extensions of our MSM are expected to contribute to this.

## Data Availability Statement

The original contributions presented in the study are included in the article/**Supplementary Material**. Further inquiries can be directed to the corresponding author.

## Author Contributions

EM, HH, XG, FC, JG, and AK designed the study. MRM designed the GRN. EM, DL, MM-H, and PR implemented the software. EM, DL, and AK carried out the simulation and analyses. All authors were involved in the interpretation of results. JG, HH, and AK supervised the study. All authors contributed to the article and approved the submitted version.

## Funding

This work is supported by a CASyM Exchange Research Grant, COSMIC (www.cosmic-h2020.eu) which has received funding from the European Union’s Horizon 2020 research and innovation program under the Marie Skłodowska-Curie grant agreement no 765158, and by the Human Frontier Science Program 570 (RGP0033/2015).

## Conflict of Interest

MR was employed by the company IBM.

The remaining authors declare that the research was conducted in the absence of any commercial or financial relationships that could be construed as a potential conflict of interest.
