# Supplementary material for: Multiscale Modeling of Germinal Center Recapitulates the Temporal Transition From Memory B Cells to Plasma Cells Differentiation as Regulated by Antigen Affinity-Based Tfh Cell Help
Source: Front Immunol. 2021 Feb 5;11:620716. doi: 10.3389/fimmu.2020.620716 (PMC7892951; doi:10.3389/fimmu.2020.620716)
Supplement: Supplementary file 1 [file DataSheet_1.pdf]

Ag saturation per FDC fragment in units of threshold. 1:constant finding proability
20
Presented Antigen per FDC
3000
Threshold Ag-concentration for binding CC (in Mol):
1e-08
B-Cell Speed (um / hr)
0.18
B-Cell Persistent Time average (hr.)
0.08
deviation of B-cell speed (um/hr.)
-1
B-Cell Persistent Time stddev (hr.)
0
Size of initial B-cell receptor pool
100
Length of BCRs
4
Lattice Chemokine Constant (um)
5
Maximum weigh of chemotaxis
10
Steepness of weight reduction with chemokine gradient (mol/l)
0.132845
Chemokine gradient of half weight (l/mol)
1.50551
Critical CXCL12 concentration for desensitization (mol)
451.654
Critical CXCL13 concentration for desensitization (mol) //(-1 for none)?????
6.02205
Critical CXCL12 concentration for resensitization (mol)
301.103
Critical CXCL13 concentration for resensitization (mol) //(-1 for none)?????
4.51654
Phase g1 of cell cycle (hr.)
2.5
Phase S of cell cycle (hr.)
1.5
Phase g2 of cell cycle (hr.)
2.5
Phase M of cell cycle (hr.)
0.5
Phase g1 of cell cycle stddev (hr.)
1
Phase S of cell cycle stddev (hr.)
1
Phase g2 of cell cycle stddev (hr.)
1
Phase M of cell cycle stddev (hr.)
1
Duration of CC collection of Antigen by serial encounters with FDC (hr.)
0.7
Standard deviation for delay to differentiation.

0
Centroblast radius (um)
2.45
Delay cell differentiation after TC selection (hr.)
6
Lattice Dimensions
3
Lattice Constant (um)
5
Time resolution (hr)
0.002
Length FDC dendrites / dx (number of positions)
8
Retained Ag is deleted in fresh CC
1
Conversion of shape space affinity to (1/mol)
5.5
Conversion of shape space affinity to (1/mol)
9.5
Exponent of the hamming distance
2
Width of gaussian affinity weight function
2.8
Initial Number Stromal cells
300
Initial Number FDCs
200
Initial Number T-cells
250
Initial Number Centroblasts
0
k_on for building immune complex (1/mol h)
36
k_off for dissociation of immune complex (in /s):
3.6
Rate of macrophage transport of dead cells (h):
0.000231049
Maximum number of residues in one dimension
9
Number of divisions of founder cells
12
stddev of Number of divisions of founder cells
0
Number of divisions of influx Bcells
6
FounderCellsDoNotMutate
0
Plasma Cell persistence time (unit)
0.16
Plasma Cell speed (unit)
0.072
Plasma Cell polarity (degrees)
-1
Probability to be selected by FDC.

0.04
% Casp3+ LZ cells per hr. used as (apoptosis rate)
0
% Casp3+ DZ cells per hr. used as (apoptosis rate)
0
Differentiation rate
0.02
p-MHC dependent division number Hill (Hill coef. n_P)
2
p-MHC dependent division number Hill (Hill coef. P_Min)
1
p-MHC dependent division number Hill (Hill coef. P_Max)
6
p-MHC dependent division number Hill (Hill coef. K_P)
9
Probability of mutation before first 24 hours
0
Probability of mutation after first 24 hours
0.5
Probability of mutation after selection (affinity dependant)
0
Affinity dependant mutation upon TC contact (affinity-exponent)
1
Probability to divide Ag assymetrically to daughter B-cell
0.72
Assymetric Distribution of Ag
1
Lattice Radius (um)
160
rate of inflow (cells/hr.)
2
Start of mutation period (hr.)
24
smoothness of stop inflow CB (hr.) (-1 = no)
6
time to stop inflow CB (hr.)
96
T-Cell Speed (um / hr.)
0.24
T-Cell Persistent Time average (hr.)
0.0705882
deviation of T-cell speed (um/sec)
-1
T-Cell Persistent Time stddev (hr.)
0
Duration of CC-Tc contact (hr.)
0.6
Minimum duration of TC-CC-polarization for CC-rescue (hr.)
0.5
Maximum duration of GC simulation (hr.)
504
Rate for differentiation of centroblasts to centrocytes
0.1
Time gap between TFHC-CC binding tests (hr.)

10
Coefficient of variation arround Polarity Index
0.04

Ag saturation per FDC fragment in units of threshold. 1:constant finding proability
20
Presented Antigen per FDC
3000
Threshold Ag-concentration for binding CC (in Mol):
1e-08
B-Cell Speed (um / hr)
0.18
B-Cell Persistent Time average (hr.)
0.08
deviation of B-cell speed (um/hr.)
-1
B-Cell Persistent Time stddev (hr.)
0
Size of initial B-cell receptor pool
100
Length of BCRs
4
Lattice Chemokine Constant (um)
5
Maximum weigh of chemotaxis
10
Steepness of weight reduction with chemokine gradient (mol/l)
0.132845
Chemokine gradient of half weight (l/mol)
1.50551
Critical CXCL12 concentration for desensitization (mol)
451.654
Critical CXCL13 concentration for desensitization (mol) //(-1 for none)?????
6.02205
Critical CXCL12 concentration for resensitization (mol)
301.103
Critical CXCL13 concentration for resensitization (mol) //(-1 for none)?????
4.51654
Phase g1 of cell cycle (hr.)
2.5
Phase S of cell cycle (hr.)
1.5
Phase g2 of cell cycle (hr.)
2.5
Phase M of cell cycle (hr.)
0.5
Phase g1 of cell cycle stddev (hr.)
1
Phase S of cell cycle stddev (hr.)
1
Phase g2 of cell cycle stddev (hr.)
1
Phase M of cell cycle stddev (hr.)
1
Duration of CC collection of Antigen by serial encounters with FDC (hr.)
0.7
Standard deviation for delay to differentiation.

0
Centroblast radius (um)
2.45
Delay cell differentiation after TC selection (hr.)
6
Lattice Dimensions
3
Lattice Constant (um)
5
Time resolution (hr)
0.002
Length FDC dendrites / dx (number of positions)
8
Retained Ag is deleted in fresh CC
1
Conversion of shape space affinity to (1/mol)
5.5
Conversion of shape space affinity to (1/mol)
9.5
Exponent of the hamming distance
2
Width of gaussian affinity weight function
2.8
Initial Number Stromal cells
300
Initial Number FDCs
200
Initial Number T-cells
250
Initial Number Centroblasts
0
k_on for building immune complex (1/mol h)
36
k_off for dissociation of immune complex (in /s):
3.6
Rate of macrophage transport of dead cells (h):
0.000231049
Maximum number of residues in one dimension
9
Number of divisions of founder cells
12
stddev of Number of divisions of founder cells
0
Number of divisions of influx Bcells
6
FounderCellsDoNotMutate
0
Plasma Cell persistence time (unit)
0.16
Plasma Cell speed (unit)
0.072
Plasma Cell polarity (degrees)
-1
Probability to be selected by FDC.

0.04
% Casp3+ LZ cells per hr. used as (apoptosis rate)
0
% Casp3+ DZ cells per hr. used as (apoptosis rate)
0
Differentiation rate
0.02
p-MHC dependent division number Hill (Hill coef. n_P)
2
p-MHC dependent division number Hill (Hill coef. P_Min)
1
p-MHC dependent division number Hill (Hill coef. P_Max)
6
p-MHC dependent division number Hill (Hill coef. K_P)
9
Probability of mutation before first 24 hours
0
Probability of mutation after first 24 hours
0.5
Probability of mutation after selection (affinity dependant)
0
Affinity dependant mutation upon TC contact (affinity-exponent)
1
Probability to divide Ag assymetrically to daughter B-cell
0.72
Assymetric Distribution of Ag
1
Lattice Radius (um)
160
rate of inflow (cells/hr.)
2
Start of mutation period (hr.)
24
smoothness of stop inflow CB (hr.) (-1 = no)
6
time to stop inflow CB (hr.)
96
T-Cell Speed (um / hr.)
0.24
T-Cell Persistent Time average (hr.)
0.0705882
deviation of T-cell speed (um/sec)
-1
T-Cell Persistent Time stddev (hr.)
0
Duration of CC-Tc contact (hr.)
0.6
Minimum duration of TC-CC-polarization for CC-rescue (hr.)
0.5
Maximum duration of GC simulation (hr.)
504
Rate for differentiation of centroblasts to centrocytes
0.1
Time gap between TFHC-CC binding tests (hr.)

10

Coefficient of variation arround Polarity Index

0.04

Ag saturation per FDC fragment in units of threshold. 1:constant finding proability
20
Presented Antigen per FDC
3000
Threshold Ag-concentration for binding CC (in Mol):
1e-08
B-Cell Speed (um / hr)
0.18
B-Cell Persistent Time average (hr.)
0.08
deviation of B-cell speed (um/hr.)
-1
B-Cell Persistent Time stddev (hr.)
0
Size of initial B-cell receptor pool
100
Length of BCRs
4
Lattice Chemokine Constant (um)
5
Maximum weigh of chemotaxis
10
Steepness of weight reduction with chemokine gradient (mol/l)
0.132845
Chemokine gradient of half weight (l/mol)
1.50551
Critical CXCL12 concentration for desensitization (mol)
451.654
Critical CXCL13 concentration for desensitization (mol) //(-1 for none)?????
6.02205
Critical CXCL12 concentration for resensitization (mol)
301.103
Critical CXCL13 concentration for resensitization (mol) //(-1 for none)?????
4.51654
Phase g1 of cell cycle (hr.)
2.5
Phase S of cell cycle (hr.)
1.5
Phase g2 of cell cycle (hr.)
2.5
Phase M of cell cycle (hr.)
0.5
Phase g1 of cell cycle stddev (hr.)
1
Phase S of cell cycle stddev (hr.)
1
Phase g2 of cell cycle stddev (hr.)
1
Phase M of cell cycle stddev (hr.)
1
Duration of CC collection of Antigen by serial encounters with FDC (hr.)
0.7
Standard deviation for delay to differentiation.

0
Centroblast radius (um)
2.45
Delay cell differentiation after TC selection (hr.)
6
Lattice Dimensions
3
Lattice Constant (um)
5
Time resolution (hr)
0.002
Length FDC dendrites / dx (number of positions)
8
Retained Ag is deleted in fresh CC
1
Conversion of shape space affinity to (1/mol)
5.5
Conversion of shape space affinity to (1/mol)
9.5
Exponent of the hamming distance
2
Width of gaussian affinity weight function
2.8
Initial Number Stromal cells
300
Initial Number FDCs
200
Initial Number T-cells
250
Initial Number Centroblasts
0
k_on for building immune complex (1/mol h)
36
k_off for dissociation of immune complex (in /s):
3.6
Rate of macrophage transport of dead cells (h):
0.000231049
Maximum number of residues in one dimension
9
Number of divisions of founder cells
12
stddev of Number of divisions of founder cells
0
Number of divisions of influx Bcells
6
FounderCellsDoNotMutate
0
Plasma Cell persistence time (unit)
0.16
Plasma Cell speed (unit)
0.072
Plasma Cell polarity (degrees)
-1
Probability to be selected by FDC.

0.04
% Casp3+ LZ cells per hr. used as (apoptosis rate)
0
% Casp3+ DZ cells per hr. used as (apoptosis rate)
0
Differentiation rate
0.02
p-MHC dependent division number Hill (Hill coef. n_P)
2
p-MHC dependent division number Hill (Hill coef. P_Min)
1
p-MHC dependent division number Hill (Hill coef. P_Max)
6
p-MHC dependent division number Hill (Hill coef. K_P)
9
Probability of mutation before first 24 hours
0
Probability of mutation after first 24 hours
0.5
Probability of mutation after selection (affinity dependant)
0
Affinity dependant mutation upon TC contact (affinity-exponent)
1
Probability to divide Ag assymetrically to daughter B-cell
0.72
Assymetric Distribution of Ag
1
Lattice Radius (um)
160
rate of inflow (cells/hr.)
2
Start of mutation period (hr.)
24
smoothness of stop inflow CB (hr.) (-1 = no)
6
time to stop inflow CB (hr.)
96
T-Cell Speed (um / hr.)
0.24
T-Cell Persistent Time average (hr.)
0.0705882
deviation of T-cell speed (um/sec)
-1
T-Cell Persistent Time stddev (hr.)
0
Duration of CC-Tc contact (hr.)
0.6
Minimum duration of TC-CC-polarization for CC-rescue (hr.)
0.5
Maximum duration of GC simulation (hr.)
504
Rate for differentiation of centroblasts to centrocytes
0.1
Time gap between TFHC-CC binding tests (hr.)

10
Coefficient of variation arround Polarity Index
0.04

Ag saturation per FDC fragment in units of threshold. 1:constant finding proability
20
Presented Antigen per FDC
3000
Threshold Ag-concentration for binding CC (in Mol):
1e-08
B-Cell Speed (um / hr)
0.18
B-Cell Persistent Time average (hr.)
0.08
deviation of B-cell speed (um/hr.)
-1
B-Cell Persistent Time stddev (hr.)
0
Size of initial B-cell receptor pool
100
Length of BCRs
4
Lattice Chemokine Constant (um)
5
Maximum weigh of chemotaxis
10
Steepness of weight reduction with chemokine gradient (mol/l)
0.132845
Chemokine gradient of half weight (l/mol)
1.50551
Critical CXCL12 concentration for desensitization (mol)
451.654
Critical CXCL13 concentration for desensitization (mol) //(-1 for none)?????
6.02205
Critical CXCL12 concentration for resensitization (mol)
301.103
Critical CXCL13 concentration for resensitization (mol) //(-1 for none)?????
4.51654
Phase g1 of cell cycle (hr.)
2.5
Phase S of cell cycle (hr.)
1.5
Phase g2 of cell cycle (hr.)
2.5
Phase M of cell cycle (hr.)
0.5
Phase g1 of cell cycle stddev (hr.)
1
Phase S of cell cycle stddev (hr.)
1
Phase g2 of cell cycle stddev (hr.)
1
Phase M of cell cycle stddev (hr.)
1
Duration of CC collection of Antigen by serial encounters with FDC (hr.)
0.7
Standard deviation for delay to differentiation.

0
Centroblast radius (um)
2.45
Delay cell differentiation after TC selection (hr.)
6
Lattice Dimensions
3
Lattice Constant (um)
5
Time resolution (hr)
0.002
Length FDC dendrites / dx (number of positions)
8
Retained Ag is deleted in fresh CC
1
Conversion of shape space affinity to (1/mol)
5.5
Conversion of shape space affinity to (1/mol)
9.5
Exponent of the hamming distance
2
Width of gaussian affinity weight function
2.8
Initial Number Stromal cells
300
Initial Number FDCs
200
Initial Number T-cells
250
Initial Number Centroblasts
0
k_on for building immune complex (1/mol h)
36
k_off for dissociation of immune complex (in /s):
3.6
Rate of macrophage transport of dead cells (h):
0.000231049
Maximum number of residues in one dimension
9
Number of divisions of founder cells
12
stddev of Number of divisions of founder cells
0
Number of divisions of influx Bcells
6
FounderCellsDoNotMutate
0
Plasma Cell persistence time (unit)
0.16
Plasma Cell speed   (unit)
0.072
Plasma Cell polarity (degrees)
-1
Probability to be selected by FDC.

0.04
% Casp3+ LZ cells per hr. used as (apoptosis rate)
0
% Casp3+ DZ cells per hr. used as (apoptosis rate)
0
Differentiation rate
0.02
p-MHC dependent division number Hill (Hill coef. n_P)
2
p-MHC dependent division number Hill (Hill coef. P_Min)
1
p-MHC dependent division number Hill (Hill coef. P_Max)
6
p-MHC dependent division number Hill (Hill coef. K_P)
9
Probability of mutation before first 24 hours
0
Probability of mutation after first 24 hours
0.5
Probability of mutation after selection (affinity dependant)
0
Affinity dependant mutation upon TC contact (affinity-exponent)
1
Probability to divide Ag assymetrically to daughter B-cell
0.72
Assymetric Distribution of Ag
1
Lattice Radius (um)
160
rate of inflow (cells/hr.)
2
Start of mutation period (hr.)
24
smoothness of stop inflow CB (hr.) (-1 = no)
6
time to stop inflow CB (hr.)
96
T-Cell Speed (um / hr.)
0.24
T-Cell Persistent Time average (hr.)
0.0705882
deviation of T-cell speed (um/sec)
-1
T-Cell Persistent Time stddev (hr.)
0
Duration of CC-Tc contact (hr.)
0.6
Minimum duration of TC-CC-polarization for CC-rescue (hr.)
0.5
Maximum duration of GC simulation (hr.)
504
Rate for differentiation of centroblasts to centrocytes
0.1
Time gap between TFHC-CC binding tests (hr.)

10

Coefficient of variation arround Polarity Index

0.04

Ag saturation per FDC fragment in units of threshold. 1:constant finding proability
20
Presented Antigen per FDC
3000
Threshold Ag-concentration for binding CC (in Mol):
1e-08
B-Cell Speed (um / hr)
0.18
B-Cell Persistent Time average (hr.)
0.08
deviation of B-cell speed (um/hr.)
-1
B-Cell Persistent Time stddev (hr.)
0
Size of initial B-cell receptor pool
100
Length of BCRs
4
Lattice Chemokine Constant (um)
5
Maximum weigh of chemotaxis
10
Steepness of weight reduction with chemokine gradient (mol/l)
0.132845
Chemokine gradient of half weight (l/mol)
1.50551
Critical CXCL12 concentration for desensitization (mol)
451.654
Critical CXCL13 concentration for desensitization (mol) //(-1 for none)?????
6.02205
Critical CXCL12 concentration for resensitization (mol)
301.103
Critical CXCL13 concentration for resensitization (mol) //(-1 for none)?????
4.51654
Phase g1 of cell cycle (hr.)
2.5
Phase S of cell cycle (hr.)
1.5
Phase g2 of cell cycle (hr.)
2.5
Phase M of cell cycle (hr.)
0.5
Phase g1 of cell cycle stddev (hr.)
1
Phase S of cell cycle stddev (hr.)
1
Phase g2 of cell cycle stddev (hr.)
1
Phase M of cell cycle stddev (hr.)
1
Duration of CC collection of Antigen by serial encounters with FDC (hr.)
0.7
Standard deviation for delay to differentiation.

0
Centroblast radius (um)
2.45
Delay cell differentiation after TC selection (hr.)
6
Lattice Dimensions
3
Lattice Constant (um)
5
Time resolution (hr)
0.002
Length FDC dendrites / dx (number of positions)
8
Retained Ag is deleted in fresh CC
1
Conversion of shape space affinity to (1/mol)
5.5
Conversion of shape space affinity to (1/mol)
9.5
Exponent of the hamming distance
2
Width of gaussian affinity weight function
2.8
Initial Number Stromal cells
300
Initial Number FDCs
200
Initial Number T-cells
250
Initial Number Centroblasts
0
k_on for building immune complex (1/mol h)
36
k_off for dissociation of immune complex (in /s):
3.6
Rate of macrophage transport of dead cells (h):
0.000231049
Maximum number of residues in one dimension
9
Number of divisions of founder cells
12
stddev of Number of divisions of founder cells
0
Number of divisions of influx Bcells
6
FounderCellsDoNotMutate
0
Plasma Cell persistence time (unit)
0.16
Plasma Cell speed (unit)
0.072
Plasma Cell polarity (degrees)
-1
Probability to be selected by FDC.

0.04
% Casp3+ LZ cells per hr. used as (apoptosis rate)
0
% Casp3+ DZ cells per hr. used as (apoptosis rate)
0
Differentiation rate
0.02
p-MHC dependent division number Hill (Hill coef. n_P)
2
p-MHC dependent division number Hill (Hill coef. P_Min)
1
p-MHC dependent division number Hill (Hill coef. P_Max)
6
p-MHC dependent division number Hill (Hill coef. K_P)
9
Probability of mutation before first 24 hours
0
Probability of mutation after first 24 hours
0.5
Probability of mutation after selection (affinity dependant)
0
Affinity dependant mutation upon TC contact (affinity-exponent)
1
Probability to divide Ag assymetrically to daughter B-cell
0.72
Assymetric Distribution of Ag
1
Lattice Radius (um)
160
rate of inflow (cells/hr.)
2
Start of mutation period (hr.)
24
smoothness of stop inflow CB (hr.) (-1 = no)
6
time to stop inflow CB (hr.)
96
T-Cell Speed (um / hr.)
0.24
T-Cell Persistent Time average (hr.)
0.0705882
deviation of T-cell speed (um/sec)
-1
T-Cell Persistent Time stddev (hr.)
0
Duration of CC-Tc contact (hr.)
0.6
Minimum duration of TC-CC-polarization for CC-rescue (hr.)
0.5
Maximum duration of GC simulation (hr.)
504
Rate for differentiation of centroblasts to centrocytes
0.1
Time gap between TFHC-CC binding tests (hr.)

10

Coefficient of variation arround Polarity Index

0.04
