# Supplementary material for: Multiscale Modeling of Germinal Center Recapitulates the Temporal Transition From Memory B Cells to Plasma Cells Differentiation as Regulated by Antigen Affinity-Based Tfh Cell Help
Source: Front Immunol. 2021 Feb 5;11:620716. doi: 10.3389/fimmu.2020.620716 (PMC7892951; doi:10.3389/fimmu.2020.620716)
Supplement: Supplementary file 2 [file DataSheet_2.docx]

**Supplementary Information**

**1. Computational model of gene regulatory network**

The gene regulatory network (GRN) involved in PC differentiation and which we used in the multiscale model comprises a pre-existing model (1). This model, comprising ordinary differential equations (ODE; Eq 1 - 3), describe the dynamics of three transcription factors (TF), i.e., BCL6 (b), BLIMP1 (p) and IRF4 (r) in terms of their dissociation constant (k), transcription (μ) and degradation rates (λ). ‘Squares’ in the equations represent Hill coefficients that enter due to the assumption of cooperative binding. The effect of the upstream BcR and CD40 signals is integrated through two separate equations (Eq 4 and 5). We embedded this model without any changes in our multiscale model. Parameter values, units and description are given in Supplementary Table 1 and were derived from literature or from fitting the model to gene expression data obtained from human GC B cells and plasma cells (see (1)). ODEs were solved using an adaptive Euler method. Several parameters are normalized by a unit of time (t_0_=4 hrs) representing the mean life time of BCL6, IRF4, and BLIMP1. In this unit the degradation rates are 1. The unit of concentration was taken as C_0_ =10^-8^M representing the average dissociation constants of IRF4 and BLIMP1 binding to its binding sites. In this unit all dissociation constants are 1. Parameter bcr_0_ represent the maximum BcR signal strength and is set to 1 in all simulations. The value of cd40 represents the constant (cd40=50, cd40=10) or affinity-based (affinity*50) CD40 signal (see main text). For further information about this model we refer to Martínez (2012). The initial values for the concentrations of BCL6, IRF4 and BLIMP1 at the start of the simulation are 11.26, 0.1, and 0.1 *10^-8^M respectively.

$$\boldsymbol{Eq 1 :}\frac{\boldsymbol{dp}}{\boldsymbol{dt}}\boldsymbol{=}\boldsymbol{\mu}_{\boldsymbol{p}}\boldsymbol{+}\boldsymbol{\sigma}_{\boldsymbol{p}}\frac{\boldsymbol{k}_{\boldsymbol{b}}^{\boldsymbol{2}}}{\boldsymbol{k}_{\boldsymbol{b}}^{\boldsymbol{2}}\boldsymbol{+}\boldsymbol{b}^{\boldsymbol{2}}}\boldsymbol{+}\boldsymbol{\sigma}_{\boldsymbol{p}}\frac{\boldsymbol{r}^{\boldsymbol{2}}}{\boldsymbol{k}_{\boldsymbol{r}}^{\boldsymbol{2}}\boldsymbol{+}\boldsymbol{r}^{\boldsymbol{2}}}\boldsymbol{-}\boldsymbol{\lambda}_{\boldsymbol{p}}\boldsymbol{p}$$

$$\boldsymbol{Eq 2 :}\frac{\boldsymbol{db}}{\boldsymbol{dt}}\boldsymbol{=}\boldsymbol{\mu}_{\boldsymbol{b}}\boldsymbol{+}\boldsymbol{\sigma}_{\boldsymbol{b}} \frac{\boldsymbol{k}_{\boldsymbol{p}}^{\boldsymbol{2}}}{\boldsymbol{k}_{\boldsymbol{p}}^{\boldsymbol{2}}\boldsymbol{+}\boldsymbol{p}^{\boldsymbol{2}}} \frac{\boldsymbol{k}_{\boldsymbol{b}}^{\boldsymbol{2}}}{\boldsymbol{k}_{\boldsymbol{b}}^{\boldsymbol{2}}\boldsymbol{+}\boldsymbol{b}^{\boldsymbol{2}}}\frac{\boldsymbol{k}_{\boldsymbol{r}}^{\boldsymbol{2}}}{\boldsymbol{k}_{\boldsymbol{r}}^{\boldsymbol{2}}\boldsymbol{+}\boldsymbol{r}^{\boldsymbol{2}}}\boldsymbol{-}\boldsymbol{(\lambda}_{\boldsymbol{b}}\boldsymbol{+ BCR)b}$$

$$\boldsymbol{Eq 3:}\frac{\boldsymbol{dr}}{\boldsymbol{dt}}\boldsymbol{=}\boldsymbol{\mu}_{\boldsymbol{r}}\boldsymbol{+}\boldsymbol{\sigma}_{\boldsymbol{r}} \frac{\boldsymbol{r}^{\boldsymbol{2}}}{\boldsymbol{k}_{\boldsymbol{r}}^{\boldsymbol{2}}\boldsymbol{+}\boldsymbol{r}^{\boldsymbol{2}}}\boldsymbol{+ CD}\boldsymbol{40-}\boldsymbol{\lambda}_{\boldsymbol{r}}\boldsymbol{r}$$

$$\boldsymbol{Eq 4:BCR=bcr}\boldsymbol{0}\frac{\boldsymbol{k}_{\boldsymbol{b}}^{\boldsymbol{2}}}{\boldsymbol{k}_{\boldsymbol{b}}^{\boldsymbol{2}}\boldsymbol{+}\boldsymbol{b}^{\boldsymbol{2}}}$$

$$\boldsymbol{Eq 5:CD}\boldsymbol{40=cd}\boldsymbol{40}\frac{\boldsymbol{k}_{\boldsymbol{b}}^{\boldsymbol{2}}}{\boldsymbol{k}_{\boldsymbol{b}}^{\boldsymbol{2}}\boldsymbol{+}\boldsymbol{b}^{\boldsymbol{2}}}$$

**Supplementary Table 1.** Parameters for the computational ODE model of the GRN. Parameters are normalized by a unit of time (t_0_) and concentration (C_0_).

| **Parameter** | **Value** | **Units** | **Description** |
| --- | --- | --- | --- |
| **μ_p_** | 10^-6^ | C_0_^/^ t_0_ | Basal transcription rate |
| **μ_b_** | 2 | C_0_^/^ t_0_ |  |
| **μ_r_** | 0.1 | C_0_^/^ t_0_ |  |
| **σ_p_** | 9 | C_0_^/^ t_0_ | Maximum induced transcription rate |
| **σ_b_** | 100 | C_0_^/^ t_0_ |  |
| **σ_r_** | 2.6 | C_0_^/^ t_0_ |  |
| **κ_p_** | 1 | C_0_ | Dissociation constant: ligand concentration that produces half of the maximum induced transcription rate |
| **κ_b_** | 1 | C_0_ |  |
| **κ_r_** | 1 | C_0_ |  |
| **λ_p_** | 1 | 1^/^ t_0_ | Degradation rate |
| **λ_b_** | 1 | 1^/^ t_0_ |  |
| **λ_r_** | 1 | 1^/^ t_0_ |  |
| **bcr0** | 1 | 1/t_0_ | Range of BCR-induced degradation of BCL6 |
| **CD40** | 0-50 | C_0_^/^ t_0_ | CD40 signal (constant: cd0=50, cd0=10, affinity-based: cd0=affinity*50) |
| **C_0_** | 10^-8^ | M | Concentration unit |
| **t_0_** | 4 | h | Time unit |

**2. Agent-based model**

*Software and parameter values*

The agent-based model (ABM) is described in more detail in Meyer-Hermann (2012) (2) and Robert (2017) (3) and references therein. The C++ software that we used for the simulations is available on request. Parameter values for the simulations listed in Supplementary files Parameters_1 to Parameters_5 for simulation 1 to 5 respectively. For a further explanation of the parameters see Robert (2017) (3).

*Affinity classes*

The discrete 4-dimensional shape space that represents affinity translates to 25 affinity classes shown in Supplementary Figure 1 (4,5). Since affinity for founder cells is initialized at a Manhattan distance (1-norm) of 4-8 the B-cells mostly assume the upper ~10 affinity classes from 10^-5^ to 1 as is shown in Supplementary Figure 2 for PCs produced in the Scenario 1, affinity-based CD40 signaling simulation (simulation 2).


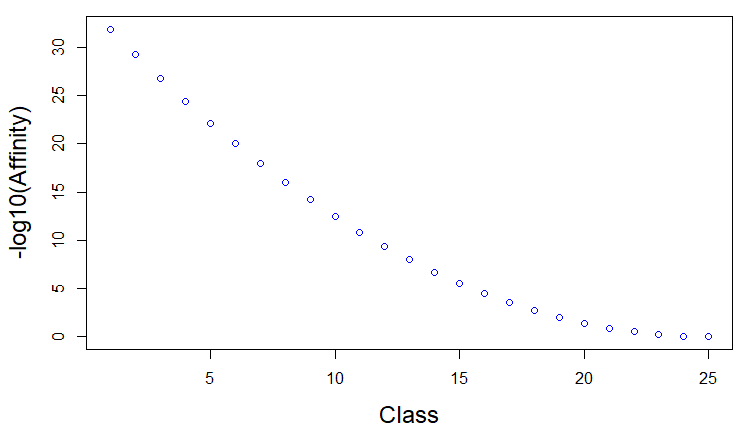


**Supplementary Figure 1:** 28 affinity classes. Affinity is represented on a logarithmic scale corresponding to affinity values of 1 to less than 10^-40^.


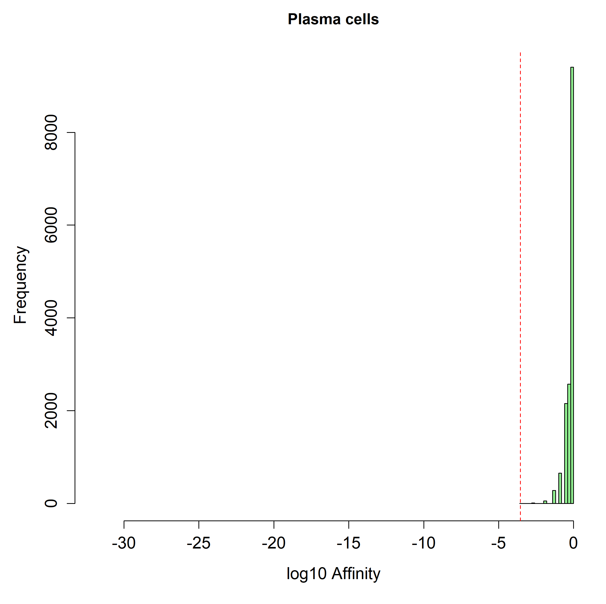


**Supplementary Figure 2:** Affinity distribution of PCs generated in Scenario 1, constant CD40=50 simulation. Only the upper affinity classes are utilized as a result of the initialization of founder cells.

**3. Single cell lineages**


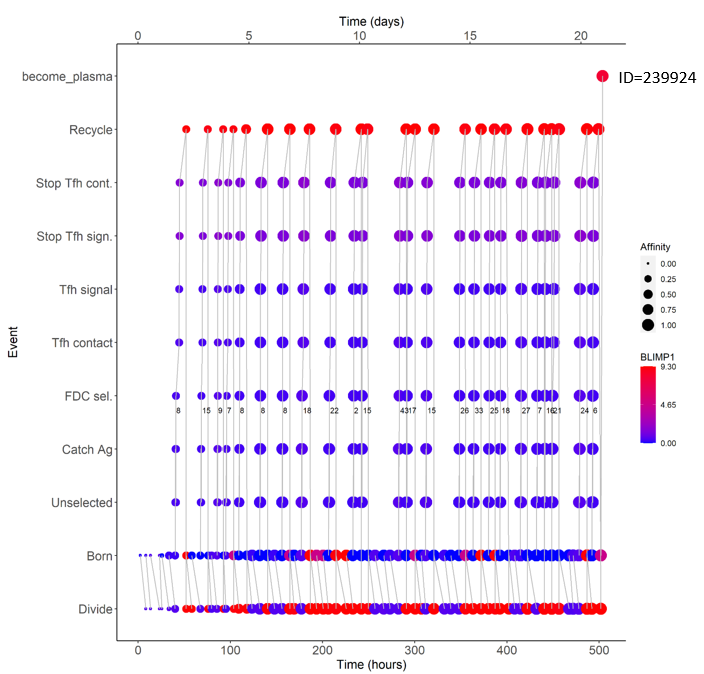


**Supplementary Figure 3:** Scenario 2, Constant CD40 signal. The PC (ID=239924) was selected and the full lineage was traced back to the initial founder cell. Note that at every cell division we obtain two daughter cells but since we trace back from the end to the beginning only one daughter cell after each division is shown. The bottom and top x-axis show the time in hours and days. The y-axis denotes the sequence of events each cell goes through unless it dies. The numbers inside the figure denote the number of attempts to bind the FDC. Size of the dots denote affinity. We observe that founder cells have a low affinity, while the final PC has a high affinity. Colors show the BLIMP1 level. In this particular lineage we observe that, as expected, the BLIMP1 level quickly goes up after Tfh interaction. At this stage the B-cell has been positively selected and will recycle to the dark zone to engage in one or more cell divisions. In this particular lineage each sequence of cell divisions ends with an asymmetric division, which results in one of the daughter cells to have no BLIMP1 preventing it from differentiating to a PC. Only after the last division at around 21 days the cell differentiates to a PC. This is shown more clearly for a smaller time interval in Supplementary Figure 4.


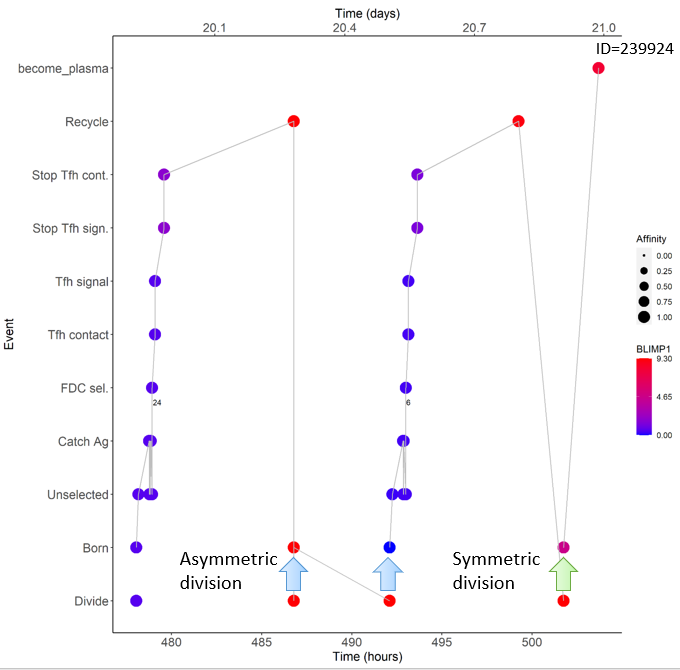


**Supplementary Figure 4:** Scenario 2, Constant CD40 signal. This shows the last part of the lineage from Supplementary Figure 3. We selected the cell (PC; ID=239924) and traced back the lineage until t=475 hours. Here, a cell with a high BLIMP1 level engages in a first asymmetric division (blue arrow; t=487) where the resulting daughter cells receives all BLIMP1. Subsequently, a second asymmetric division follows (blue arrow; t=493) where the daughter cell does not receive any BLIMP1 and prevents the cell from differentiating to a PC. Only in the final symmetric division (t=502) the BLIMP1 level of the daughter cells stays high enough to enable PC differentiation.


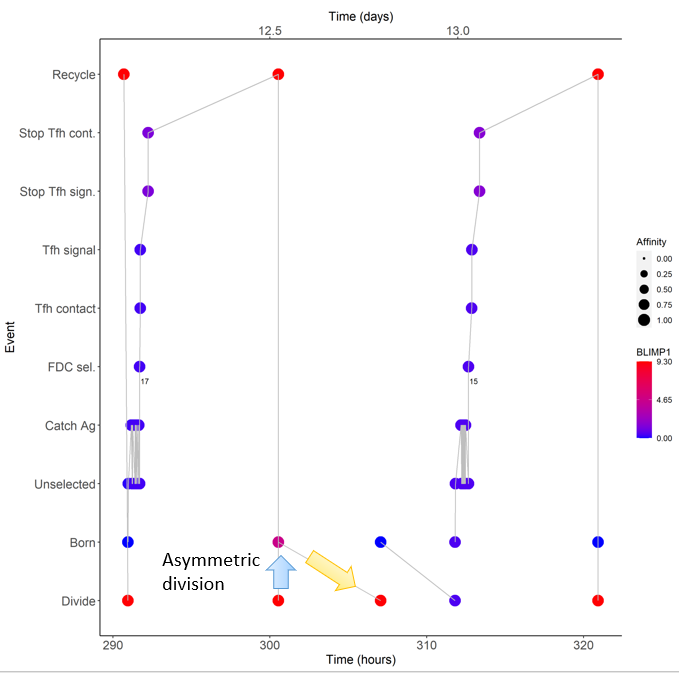


**Supplementary Figure 5:** Scenario 2, Constant CD40 signal. This shows part of the lineage Supplementary Figure 3. Here the lineage is shown from t=290 to t=325 hours. We observe that a cell with a high BLIMP1 level symmetrically divides (t=301) resulting in a daughter cell with an intermediate BLIMP1 level (blue arrow). However, within the time of the next division the BLIMP1 levels returns to its high equilibrium value (BLIMIP1+; yellow arrow), after which an asymmetric division at t=307) results in a daughter cells without any BLIMP.

**4. Cell type definitions**

**Supplementary Table 2.** Definition of cell types. PC=plasma cell, MBC= memory B-cell, PB=plasmablast, CB=centroblast, CC=centrocyte, NA=not applicble. In Scenario 1 simulations Ag+ cells (after asymmetric division) become and output cell, which are divided in PCs and MBCs based on post-simulation inspection of BLIMP1 level. In Scenario 2 simulations the BLIMP1 level is used to decide if a cell differentiates to an output cell. See main text (method section) for further details.

|  |  | **Scenario 1** | | **Scenario 2** | |
| --- | --- | --- | --- | --- | --- |
|  |  | **BLIMP1+** | **BLIMP1-** | **BLIMP1+** | **BLIMP1-** |
| OUTPUT CELL | **Ag+** | PC | MBC | PC | MBC |
| NOT OUTPUT CELL | **Ag+** | PB | CB | PB | CB |
| OUTPUT CELL | **Ag -** | NA | NA | PC | NA |
| NOT OUTPUT CELL | **Ag-** | CB/CC | CB/CC | PB | CB/CC |

The following figures (Supplementary Figure 6,7 and 8) give three examples of how different cell types are annotated.


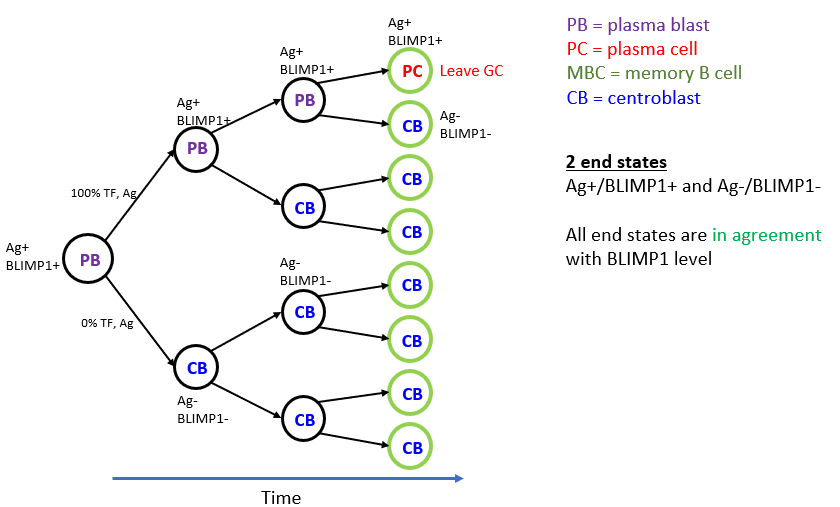


**Supplementary Figure 6:** Scenario 1, Ag decision rule. This figure shows 3 cell divisions that are all asymmetric. The parent cell is Ag+ (by definition) and BLIMP1+. As a result of asymmetric division, the Ag and BLIMP1 is inherited by only one of the daughter cells (Ag+/BLIMP1+) while the other daughter cell becomes Ag-/BLIMP1-. After 3 divisions we are left with 8 cells in only two different states. In this scenario only the Ag+ cells become output cells (a single cell in this example), which is annotated as a PC. This is in agreement (green circle) with the PC definition based on the BLIMP1 level, i.e., BLIMP1+. The 7 other cells are CBs. The three ancestors of the PC are annotated as a PB.


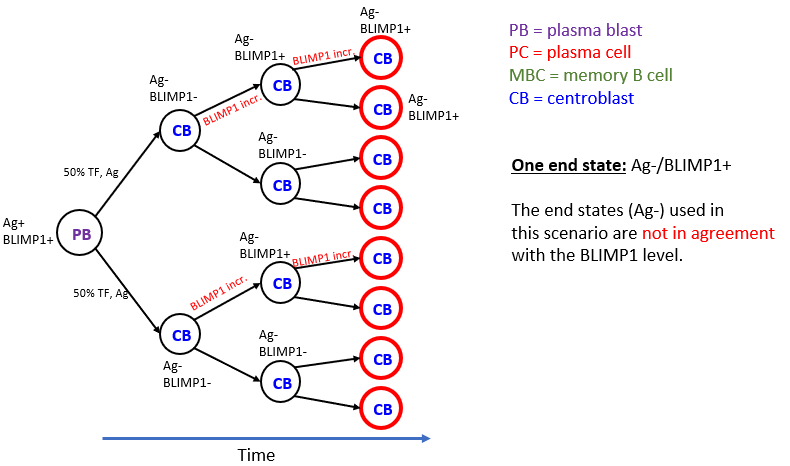


**Supplementary Figure 7:** Scenario 1, Ag decision rule. This figure shows 3 cell divisions that are all symmetric. The parent cell is Ag+ (by definition) and BLIMP1+ and annotated as a PB since it is Ag+/BLIMP1+ although it eventually does not become an output cell. Due to symmetric division both daughter cells receive 50% of the Ag and BLIMP1 and, therefore, become Ag-/BLIMP1- cells. However, since BLIMP1 (and IRF4) are still at a relatively high they will quickly return to a high BLIMP1 level (see Supplementary Figure 3 for an example) due to the bistable nature of the GRN. Consequently, in Scenario 1 no output cells result (all cells are Ag-) while considering the BLIMP1 level, all these cells should be PCs.


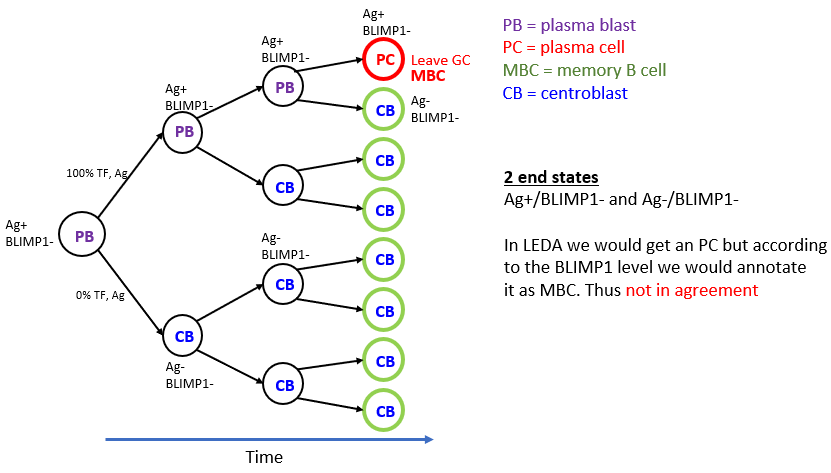


**Supplementary Figure 8:** Scenario 1, Ag decision rule. This figure shows 3 cell divisions that are all asymmetric. The parent cell is Ag+ (by definition) and BLIMP1- and annotated as a PB since it leads to an PC (Ag+ output cell). In Scenario 2 simulations we would have denoted the output cell (PC) as an MBC because it has a low BLIMP1 level and therefor it is not in agreement with the definition used in LEDA.

**5. Cell counts**

**Supplementary File Counts_and_Percentages.xlsx** comprises cell counts and calculated percentages from the simulations.

**6. Results**


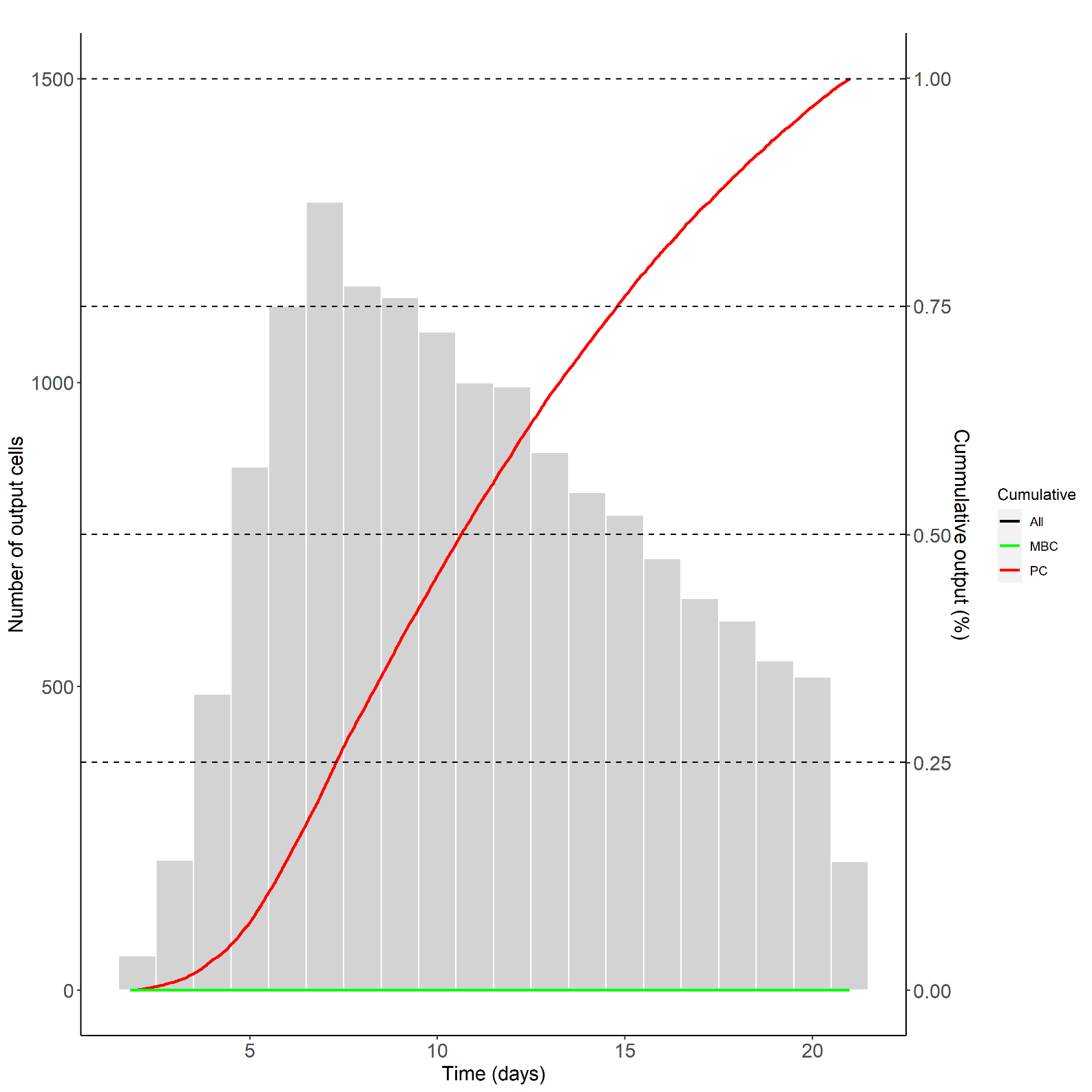


**Supplementary Figure 9**. Scenario 1, Constant cd40 (cd40=50). (Cumulative) number of output cells. Histogram represents the number of output cells per day. Red and green line represent the cumulative number of PCs and MBCs respectively. No MBCs are produced in this simulation and, therefore, the red and black line coincide. About 76% of the PCs are produced after the peak of output cells at day 7. Note: percentages of PCs and MBCs are calculated relative to the total number of PCs and MBCs respectively (not relative to the total number of output cells).


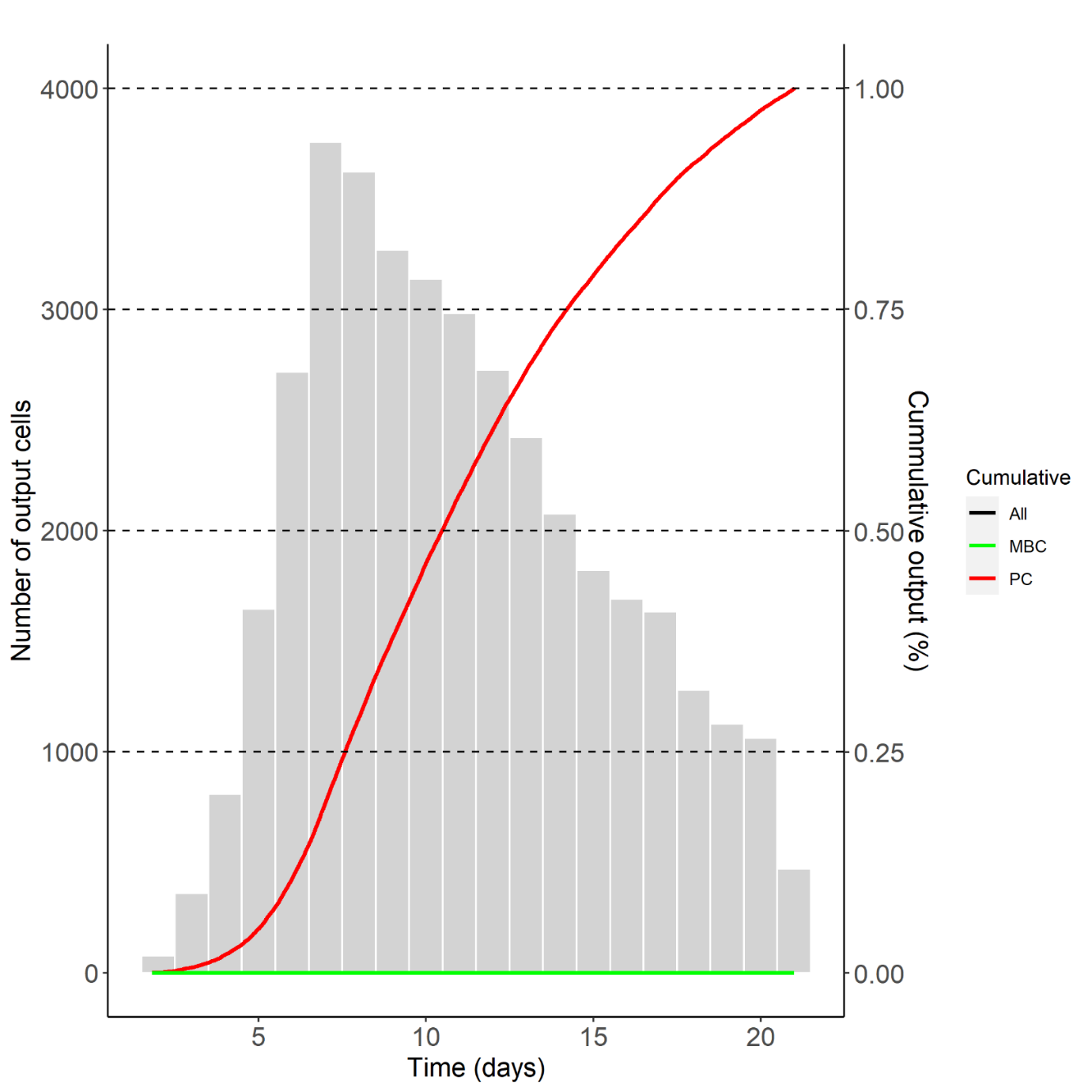


**Supplementary Figure 10**. Scenario 2, Constant cd40 (cd50=50). (Cumulative) number of output cells. Histogram represents the number of output cells per day. Red and green line represent the cumulative number of PCs and MBCs respectively. No MBCs are produced in this simulation and, therefore, the red and black line coincide. About 72% of the PCs are produced after the peak at day 7. Note: percentages of PCs and MBCs are calculated relative to the total number of PCs and MBCs respectively (not relative to the total number of output cells).


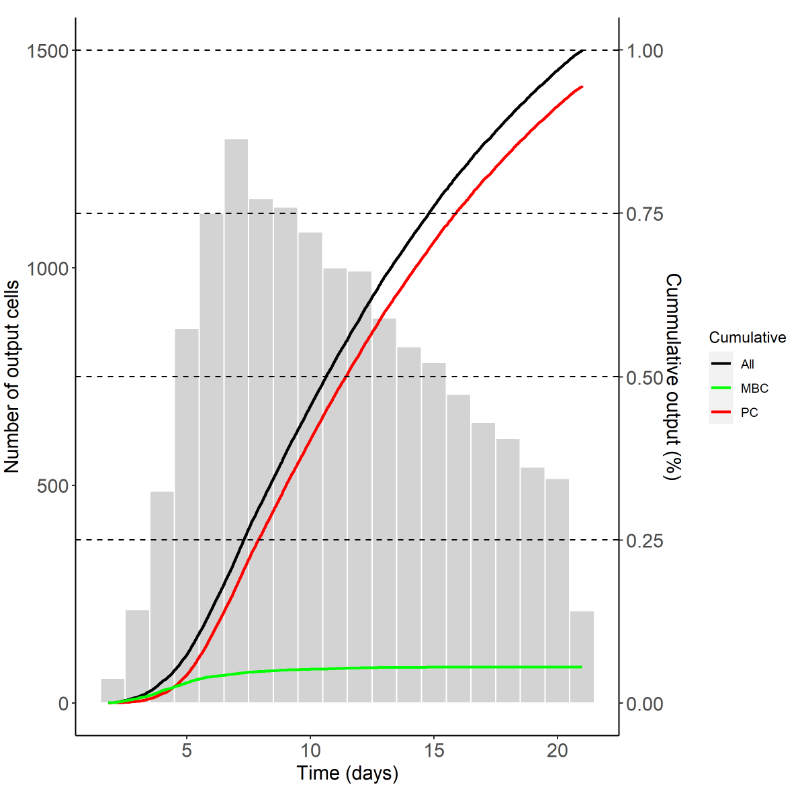


**Supplementary Figure 11**. Scenario 1, Affinity-based cd40. (Cumulative) number of output cells. Histogram represents the number of output cells per day. Red and green line represent the cumulative number of PCs and MBCs respectively. About 76% of the PCs are produced after the peak at day 7. 85% of the MBCs are produced prior to day 7. Note: percentages of PCs and MBCs are calculated relative to the total number of PCs and MBCs respectively (not relative to the total number of output cells).


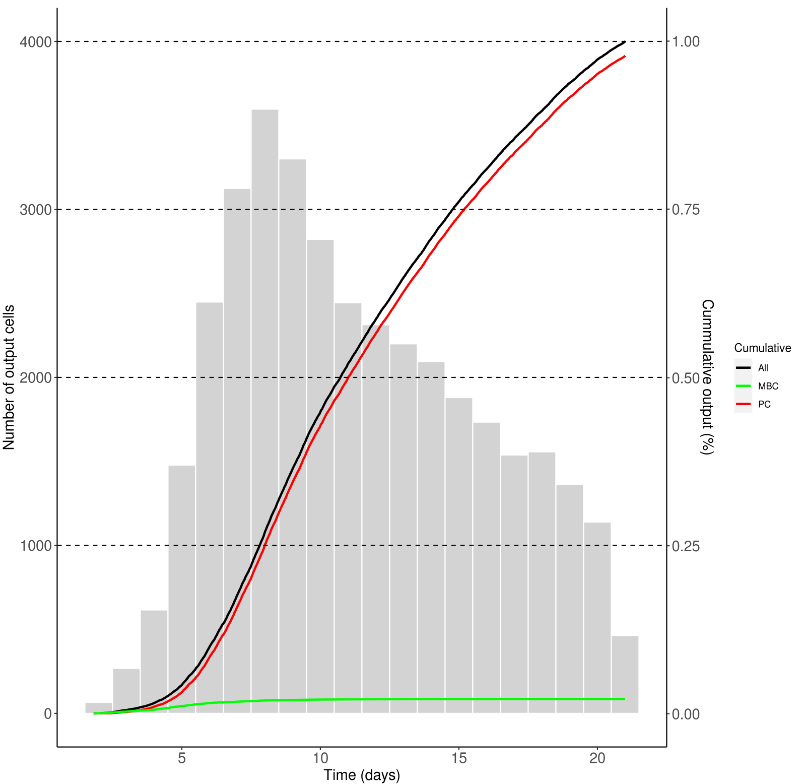


**Supplementary Figure 12**. Scenario 2, Affinity-based cd40. (Cumulative) number of output cells. Histogram represents the number of output cells per day. Red and green line represent the cumulative number of PCs and MBCs respectively. About 75% of the PCs are produced after the peak at day 8. 89% of the MBCs are produced prior to day 8. Note: percentages of PCs and MBCs are calculated relative to the total number of PCs and MBCs respectively (not relative to the total number of output cells).


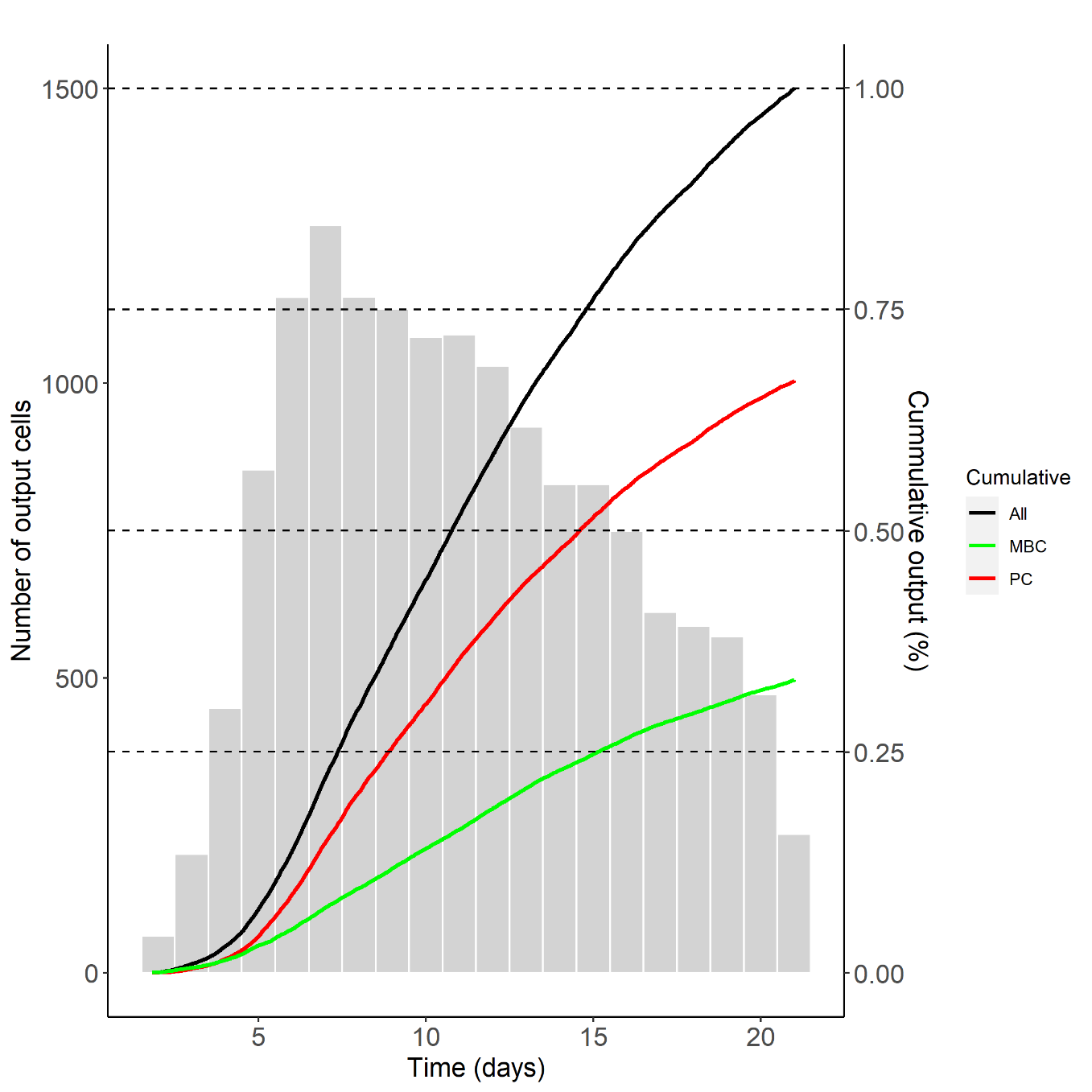


**Supplementary Figure 13**. Scenario 2, Constant cd40 (cd40=10). (Cumulative) number of output cells. Histogram represents the number of output cells per day. Red and green line represent the cumulative number of PCs and MBCs respectively. About 73% of the PCs are produced after the peak at day 8. Only 26% of MBCs are produced prior to the peak at day 8. Note: percentages of PCs and MBCs are calculated relative to the total number of PCs and MBCs respectively (not relative to the total number of output cells).


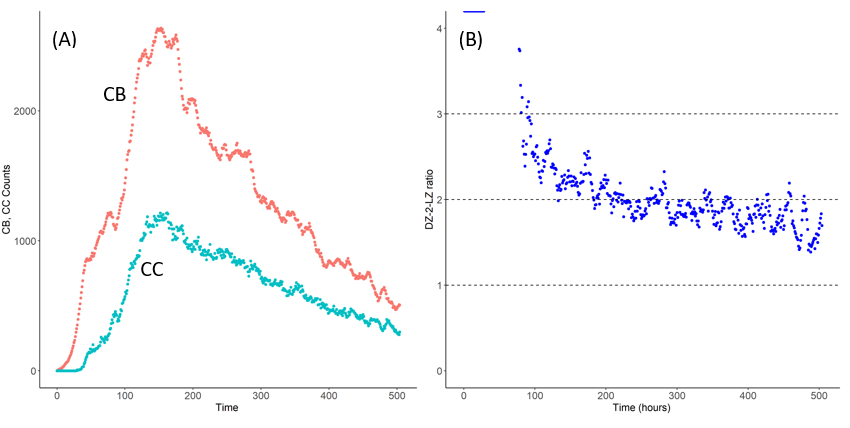


**Supplementary Figure 14**. Scenario 2, Constant cd40 (cd40=10). Overall GC dynamics. (A) CB and CC counts. (B) DZ-2-LZ ratio


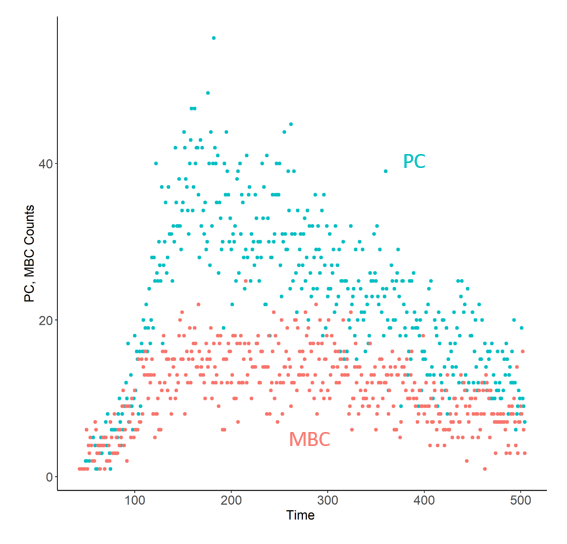


**Supplementary Figure 15**. Scenario 2, Constant cd40 (cd40=10). Number of PCs and MBCs generated during the GC reaction.

**7. Variation in cell dynamics in repeated simulations**


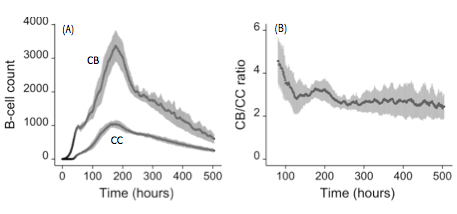


**Supplementary Figure 16.** Scenario 2, Constant (cd40=50). Overall GC dynamics. (A) CB and CC counts. (B) DZ-to-LZ ratio. Mean and standard deviation of 30 different random seeds are shown.


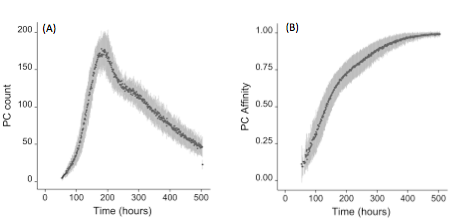


**Supplementary Figure 17**. Scenario 2, Constant (cd40=50). (A) Number of PCs and (B) Affinity of PCs generated during the GC reaction. No MBCs were generated. Mean and standard deviation of 30 different random seeds are shown.

**
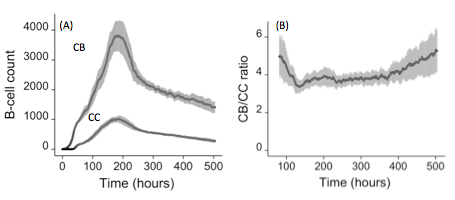
**

**Supplementary Figure 18.** Scenario 2, affinity-dependent (cd40=50*affinity).Overall GC dynamics. (A) CB and CC counts. (B) DZ-to-LZ ratio. Mean and standard deviation of 30 different random seeds are shown.


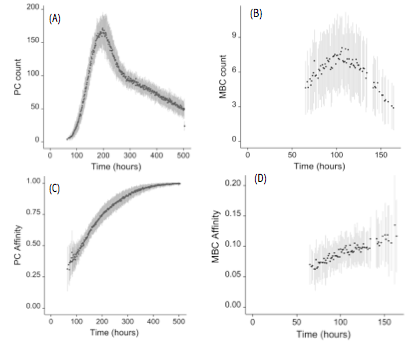


**Supplementary Figure 19.** Scenario 2, affinity-dependent (cd40=5*affinity). (A) Number of PCs, (B) Number of MBCs, (C) Affinity of PCs and (D) Affinity of MBCs generated during the GC reaction. Mean and standard deviation of 30 different seeds are shown.

**Supplementary Table 3.** Variability in calculated percentages based on 30 repetitions of Simulation 3 (Scenario 2, constant CD40 signal). Avg=average, sd=standard deviation, se=standard error, min=minimum, max=maximum, Paper=value refered to in the main text.

| **Scenario 2** | **Constant CD40 signal** | |  | **CD40=50** |  |  |  |
| --- | --- | --- | --- | --- | --- | --- | --- |
|  |  | **BLIMP1+** |  |  |  |  |  |
|  |  | **avg (%)** | **sd** | **se** | **min** | **max** | **Paper** |
| OUTPUT CELL | Ag+ | 4.3 | 0.2 | 0.0 | 4.0 | 4.7 | 4.7 |
| NOT OUTPUT CELL | Ag+ | 17.6 | 0.4 | 0.0 | 16.5 | 18.3 | 18.3 |
| OUTPUT CELL | Ag- | 9.2 | 0.1 | 0.0 | 8.9 | 9.4 | 9.2 |
| NOT OUTPUT CELL | Ag- | 9.8 | 0.2 | 0.0 | 9.2 | 10.1 | 9.3 |
|  |  | **BLIMP1-** |  |  |  |  |  |
|  |  | **avg (%)** | **sd** | **se** | **min** | **max** | **Paper** |
| OUTPUT CELL | Ag+ | 0.0 | 0.0 | 0.0 | 0.0 | 0.0 | 0.00 |
| NOT OUTPUT CELL | Ag+ | 0.0 | 0.0 | 0.0 | 0.0 | 0.1 | 0.05 |
| OUTPUT CELL | Ag- | 0.0 | 0.0 | 0.0 | 0.0 | 0.0 | 0.00 |
| NOT OUTPUT CELL | Ag- | 59.1 | 0.5 | 0.0 | 58.1 | 60.7 | 58.31 |

**Supplementary Table 4.** Variability in calculated percentages based on 30 repetitions of Simulation 4 (Scenario 2, affinity-based CD40 signal). Avg=average, sd=standard deviation, se=standard error, min=minimum, max=maximum, Paper=value refered to in the main text.

| **Scenario 2** | **Affinity-based CD40 signal** | | |  |  |  |  |
| --- | --- | --- | --- | --- | --- | --- | --- |
|  |  | **BLIMP1+** |  |  |  |  |  |
|  |  | **avg (%)** | **sd** | **se** | **min** | **max** | **Paper** |
| OUTPUT CELL | Ag+ | 3.8 | 0.3 | 0.0 | 3.2 | 4.4 | 4.1 |
| NOT OUTPUT CELL | Ag+ | 15.6 | 0.9 | 0.0 | 14.0 | 17.3 | 16.6 |
| OUTPUT CELL | Ag- | 8.3 | 0.3 | 0.0 | 7.6 | 8.8 | 8.6 |
| NOT OUTPUT CELL | Ag- | 9.1 | 0.2 | 0.0 | 8.6 | 9.4 | 9.1 |
|  |  | **BLIMP1-** |  |  |  |  |  |
|  |  | **avg (%)** | **sd** | **se** | **min** | **max** | **Paper** |
| OUTPUT CELL | Ag+ | 0.2 | 0.0 | 0.0 | 0.2 | 0.3 | 0.28 |
| NOT OUTPUT CELL | Ag+ | 6.7 | 3.3 | 0.0 | 2.5 | 14.2 | 3.53 |
| OUTPUT CELL | Ag- | 0.0 | 0.0 | 0.0 | 0.0 | 0.0 | 0.00 |
| NOT OUTPUT CELL | Ag- | 56.2 | 1.7 | 0.0 | 52.0 | 58.6 | 57.78 |

Note that variability is small for all percentages except for BLIMP1-/Ag+/No output cell. However, this Ag+/BLIIMP1- cell is not of particular interest in our analyses. Similarly, the Ag-/BLIMP1- has a somewhat larger standard deviation (1.7%) but also this cell type we do not explicitly consider.


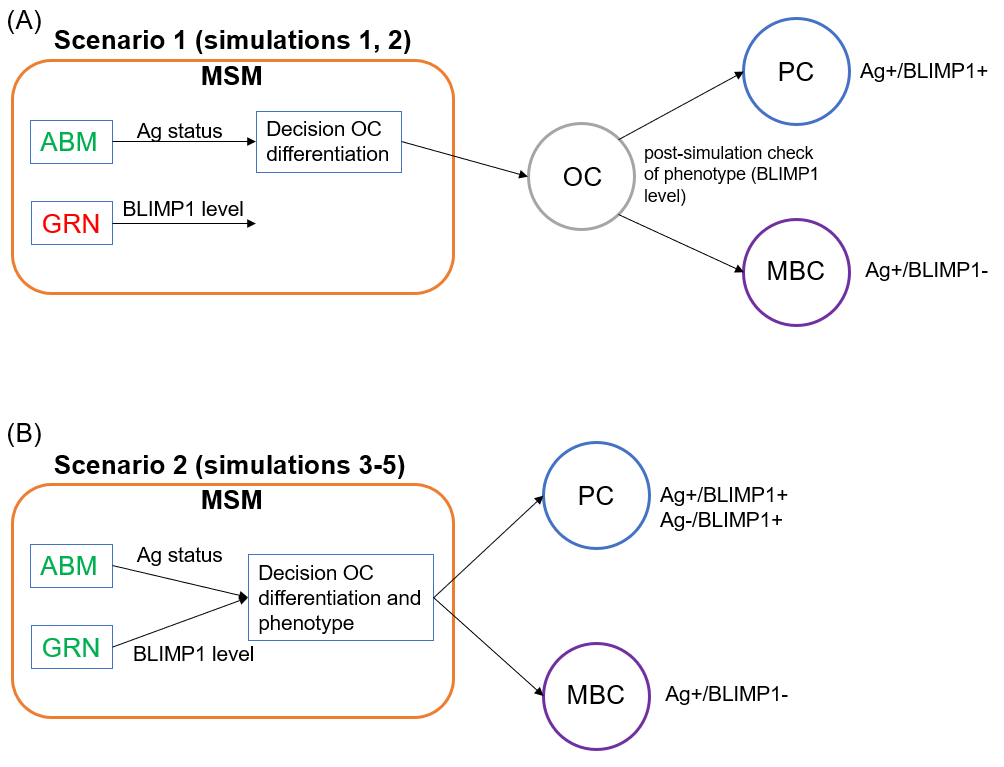


**Supplementary Figure 20.**  Cell-fate decision and phenotype definition of output cells (OC) in Scenario 1 and Scenario 2 simulations. (A) In Scenario 1 the decision for OC differentiation is made at the ABM level (cell level) based on Ag status (Ag-retaining cells after asymmetric division will differentiate to an OC) but BLIMP1 level determines final phenotype (PC or MBC) after production of the OCs. (B) In Scenario 2 both the Ag status and BLIMP1 level are used to decide for OC differentiation and phenotype. Note, only in Simulations 1 and 3 no MBCs are produced.

**References**

1. Martínez MR, Corradin A, Klein U, Álvarez MJ, Toffolo GM, Di Camillo B, et al. Quantitative modeling of the terminal differentiation of B cells and mechanisms of lymphomagenesis. Proc Natl Acad Sci U S A. 2012;109(7):2672–7.

2. Meyer-Hermann M, Mohr E, Pelletier N, Zhang Y, Victora GD, Toellner KM. A theory of germinal center b cell selection, division, and exit. Cell Rep. 2012;2(1):162–74.

3. Robert PA. How to simulate a Germinal Center. Methods Mol Biol. 2017;1623.

4. Meyer-Hermann M, Deutsch A, Or-Guil M. Recycling Probability and Dynamical Properties of Germinal Center Reactions. J Theor Biol [Internet]. 2001;210(3):265–85. Available from: http://www.sciencedirect.com/science/article/pii/S0022519301922970

5. Perelson AS, Oster GF. Theoretical studies of clonal selection minimal antibody repertoire size and reliability of self non self discrimination. J Theor Biol. 1979;81(4):645–70.
